# Apoptotic signatures allow early and rapid screening of drug-induced liver injury to accelerate drug discovery

**DOI:** 10.1038/s43856-025-01306-7

**Published:** 2025-12-24

**Authors:** John Hellgren, Bhavik Chouhan, Aydar Uatay, Ramy Elgendy, Julia Lindgren, Naoko Toki, Alessandro Bonetti, Aditi Chaudhari, Kenneth Pryde, Patrik Andersson, Marie Kalm, Fredrik Karlsson, Johanna Sagemark, Dominic P. Williams, Jennifer Y. Tan, Bino John, John Gallon

**Affiliations:** 1 Imaging & Data Analytics, Clinical Pharmacology & Safety Sciences, R&D, AstraZeneca, Gothenburg, Sweden; 2 Safety Sciences, Clinical Pharmacology & Safety Sciences, R&D, AstraZeneca, Gothenburg, Sweden; 3 Clinical Pharmacology & Quantitative Pharmacology, Clinical Pharmacology & Safety Sciences, R&D, AstraZeneca, Cambridge, United Kingdom; 4 Translational Genomics, Centre for Genomics Research, Discovery Sciences, R&D, AstraZeneca, Gothenburg, Sweden; 5 Discovery Biology, Discovery Sciences, R&D, AstraZeneca, Gothenburg, Sweden; 6 Safety Sciences, Clinical Pharmacology & Safety Sciences, R&D, AstraZeneca, Waltham, MA USA; 7 Data Sciences & Quantitative Biology, Discovery Sciences, R&D, AstraZeneca, Gothenburg, Sweden; 8 Biometrics, Respiratory & Immunology, R&D, AstraZeneca, Gothenburg, Sweden; 9 Safety Sciences, Clinical Pharmacology & Safety Sciences, R&D, AstraZeneca, Cambridge, United Kingdom; 10 Imaging & Data Analytics, Clinical Pharmacology & Safety Sciences, R&D, AstraZeneca, Cambridge, United Kingdom; 11 Imaging & Data Analytics, Clinical Pharmacology & Safety Sciences, R&D, AstraZeneca, Boston, MA USA

**Keywords:** Drug screening, Dynamical systems, Transcriptomics, High-throughput screening, Drug discovery

## Abstract

**Background:**

Early detection of drug-induced liver injury (DILI) during drug development is crucial for reducing drug attrition and ensuring the safety of patients. A versatile, biologically interpretable, and dose-dependent screening approach is therefore needed to inform early stop/go decisions and therapeutic margins.

**Methods:**

We have developed AEGIS (Apoptotic Effector Genes In Safety), a preclinical DILI risk screening and prioritization tool that quantifies dose dependent perturbation of apoptosis-regulating transcription factors from transcriptomics data. We profiled transcriptomic responses after short exposures across primary human hepatocytes (PHH), HepG2/C3A cells, RAW 264.7 cells, and an acute Balb/c mouse study. From these profiles, AEGIS provides quantitative risk scores to rank and prioritize compounds and exposures.

**Results:**

Here we show that AEGIS distinguishes compounds with different degree of DILI concern, achieving 86% specificity, 75% sensitivity and 90% precision in PHHs. We demonstrate versatility in data type usage and clinical translation of AEGIS with accurate predictions across species, in vitro and in vivo models, and therapeutic modalities. In addition, we apply AEGIS in a precision medicine context during drug-development within the pharmaceutical industry and investigate the contribution of underlying liver disease on DILI severity. Our findings indicate that cells from patients with metabolic dysfunction-associated steatotic liver disease (MASLD) develop more severe DILI from treatment with troglitazone, aligning with preclinical observations.

**Conclusions:**

Using AEGIS early in drug discovery exemplifies a more efficient approach to identify and mitigate potential safety concerns. This can reduce the need for animal testing, and accelerates drug discovery, ultimately providing the right medicines to patients more quickly.

## Plain language summary

Some medicines can harm the liver, leading to side effects in people taking the drug, failure of drugs being trialed for use in patients, and delayed new treatments. We developed AEGIS, a screening tool that looks at how cells respond to drugs to spot early warning signs that a drug may injure liver cells. We tested it across human liver cells and animals. It consistently identified drugs that might damage the liver at realistic doses. It also showed that cells from people with fatty liver disease may be more vulnerable to liver injury. Using AEGIS early in development could help remove unsafe medicines sooner, reduce animal use, and bring safer treatments to patients faster.

## Abstract (web summary)

Hellgren et al. develop AEGIS, a transcriptomics-based tool that quantifies apoptosis-linked gene signatures to assess drug-induced liver injury risk across doses, in vitro models, species, and modalities. AEGIS ranks compounds by liver risk, translates from in vitro to in vivo, and can accelerate safer drug development.

## Introduction

Drug development is a complex endeavour with high failure rates due to safety concerns^1^, resulting in patients having to wait longer for the right treatment. Despite many advancements in drug development technologies^2^, the success rate remains at approximately 8%^3^, involving several crucial decision-making steps. The concept of ‘failing early’ has become increasingly important to ensure patient safety, and conducting clinical trials remains the most expensive phase of the drug development process^4^. However, around 40% of drug programs fail during phase I trials due to clinical safety concerns^1^. Establishing quantitative risk assessments and early detection of preclinical adverse events allows for either the mitigation of these safety concerns or stopping compounds before they reach clinical trials. Drug-induced liver injury (DILI) is a major cause of drug attrition^5^, accounting for 18% of post-market withdrawn drugs^6^. DILI can be classified into two different types: intrinsic and idiosyncratic DILI^7^. Intrinsic DILI is dose-dependent and predictable, whereas idiosyncratic DILI is not dose-dependent and is difficult to predict^7^.

Given the risk of inducing liver injury, it is vital to establish high-throughput and cost-effective screening platforms that can assess many compounds quickly, enabling robust stop/go decisions and guiding the redesign of compound series to maximise liver safety profiles. Safety assessment in animal models is the final stage of the discovery process, aimed at ensuring that compounds entering clinical trials are safe. However, there is limited translatability between species, as the ability to detect clinical DILI in a preclinical species may be as low as 30% sensitivity^8^. Therefore, better predictive tools are needed in the preclinical discovery phase.

The generation of gene expression data during drug discovery can provide further insights into the risks and mechanisms of DILI^9^. Additionally, this data allows the construction of predictive models that can complement or replace DILI predictions made using bioactivity data. Microarray and targeted microarray L1000^10^ data have been successfully utilised to build multiple predictive models of DILI, with specificity and sensitivity ranging between 71-100% and 58-86 %, respectively^11–15^. However, care must be taken to avoid data leakage and overfitting when evaluating these models, as these issues can lead to overestimated performance. Furthermore, machine learning models can often be perceived as black boxes with limited interpretability, and their use is often restricted to a specific data type generated in a particular model. Therefore, the field lacks a model that is biologically interpretable, applicable to different data sources, and drug-modality agnostic.

In the era of precision medicine, the transition from binary DILI predictions for compounds to dose-dependent predictions is important, enabling the definition of therapeutic margins when used alongside the therapeutic maximum serum concentration (C_max_), which can be adjusted for different patient groups. Patients with pre-existing liver conditions are suspected to have increased risk for DILI^16^ and should be considered during safety assessment. For example, metabolic dysfunction-associated steatotic liver disease (MASLD), formerly non-alcoholic fatty liver disease (NAFLD)^17^, has 25% world-wide prevalence^18^ and is connected to pharmacokinetic changes in the liver^19,20^

Here, we have developed a DILI risk estimation tool that is based on fundamental biology: the perturbation of apoptosis-regulating transcription factors; a defining feature of DILI^21^. Our tool, Apoptotic Effector Genes In Safety (AEGIS), calculates a dose-dependent DILI score from the change in expression of the target genes of these transcription factors in response to drug treatment. The minimal parameter tuning used in AEGIS limits the risk of overfitting, and we show that AEGIS can be applied across species, multiple transcriptomics technologies, and treatment modalities with high prediction accuracies. Throughout this work, we use AEGIS to assess compounds and exposures based on risk of intrinsic DILI, while risk of idiosyncratic DILI is outside the scope of this paper. Furthermore, we applied AEGIS in a precision medicine context by comparing the DILI score in cells from patients with MASLD and cells from healthy patients. AEGIS predicts that cells from patients with MASLD develop more severe DILI from treatment with the known hepatotoxic compound troglitazone, suspected to cause more severe DILI in MASLD preclinically^22–24^, compared to cells from healthy patients. AEGIS accelerates drug discovery by ranking compounds in terms of safety, guiding the decision-making processes. AEGIS offers dose-dependent predictions, supports various data types, is applicable across species and in vitro models, and is drug-modality agnostic. This makes AEGIS a valuable tool for providing safer medicines to patients, quicker and at a lower cost.

## Methods

### Material

Cryopreserved PHH were obtained from either BioIVT (Baltimore, MD, USA and Woburn, MA, USA) or LifeNet Health (Virginia Beach, VA, USA). Additional information about the procurement of human donor samples is stated in Supplementary Methods. HepG2/C3A cells were obtained from American Type Culture Collection (ATCC, CRL-3581). Foetal Bovine Serum (FBS), for the liver spheroids and HepG2/C3A, was obtained from Pepparedsleden 1, Mölndal, Sweden. All other reagents, Williams E medium (W1878-500ML), Dulbecco’s Modified Eagle Medium (DMEM, 31966-021), GlutaMAX (35050-061), HEPES (15630-056), sodium pyruvate 100 mM (11360-039), MEM non-essential amino acids (1x, 11140-035), heat-inactivated FCS (Gibco 10500-064), Insulin-transferrin-selenium (100x, 51300044), cryopreserved hepatocyte recovery medium (CM7000), primary hepatocyte thawing and plating supplements (CM3000), primary hepatocyte maintenance supplements (CM4000) and L-glutamine–penicillin–streptomycin solution (G6784) were obtained from Thermo Fisher Scientific. Promega CellTiter-Glo ®Luminescent Cell Viability Assay kit was obtained from Promega, Madison, WI, USA. Corning® Matrigel® Basement Membrane Matrix (356237), Collagen I-coated 96-well plate (354407), Collagen I-coated 384-well plate (354667), Ultra-low adhesion (ULA) 96-well (7007), and 384-well (3830) plates were obtained from Corning. Ninety-six-well PP-V-bottom plates were from Griner (651201). All pharmaceutical drugs were provided by AstraZeneca Internal Compound Management, AstraZeneca R&D, Mölndal, Sweden, Sigma-Aldrich, St. Louis, MO, USA or Selleck Chemicals GmbH, Cologne, Germany.

### Cell culture

#### Liver spheroid preparation and cell seeding

Liver spheroids were thawed, seeded and cultured up until day 7 as detailed by ref. ^25^ with the modification that non-parenchymal cells were excluded in the incubations. PHH were seeded at 1500 hepatocytes/well and 100 µl/well into 96-well ULA plates and 80 µl/well into 384-well ULA.

#### HepG2/C3A preparation and cell seeding

Cells were thawed and seeded in DMEM glucose media containing 10% FBS, 1% GlutaMAX, 0.5% HEPES and 1% NaPy into collagen I coated 384-well plate at 3000 cell/well and placed in incubator at 37 °C overnight for 24 h.

#### 2D liver preparation and cell seeding

Cryopreserved PHH were thawed in CM7000 media at 37 °C, pelleted, resuspended and counted. Cell suspension was prepared in Williams E media supplemented with CM3000 and plated in a collagen I-coated 96-well plate at 70,000 cells/well. Plates were placed in an incubator at 37 °C for 4–6 h for cell attachment. Afterwards, the media was removed and replaced with media containing Williams E supplemented with CM4000 and Matrigel at 0.25 mg/ml, and incubated overnight for 18 h.

The list of human donors used in each figure are shown in Supplementary Data 1.

#### RAW 264.7 cell preparation and seeding

RAW 264.7 cells were cultured at a seeding density of 18,000 cells/well in a 96-well PP-V-bottom plate. The cells were cultured in DMEM medium with 4.5 g/L glucose, GlutaMax, NaPy, supplemented with 10% of heat-inactivated FCS and 1x MEM non-essential amino acids. After culturing, cells were treated for 24 h at 37 °C and 5% CO_2_ with ASO concentrations of 5, 0.56 and 0.06 µM.

### Compounds and DILI labels

**Fig. 1:**
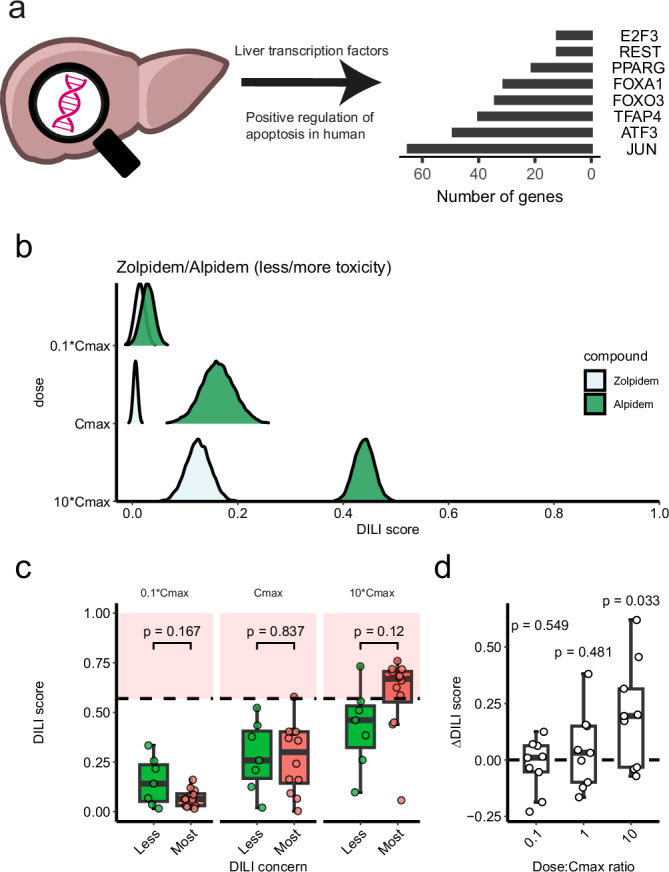
AEGIS workflow and translatability of DILI score to human clinical outcomes. **a** Eight transcription factors that are expressed in the liver were selected based on their implication in the positive regulation of apoptosis in humans. Their target genes were combined into a gene signature containing 241 genes. This signature is applied to transcriptomics data generated from compound treatment either in vitro or in vivo. **b** An example output from AEGIS applied to RNA-seq data from treatment of primary human hepatocytes with zolpidem and alpidem at doses of 0.1-, 1- and 10-times C_max_. The resulting DILI score reflects the fraction of perturbation of apoptotic pathways (0 no perturbation and 1 full perturbation). Zolpidem is connected to an *ambiguous* *DILI* *concern*, and alpidem to *most* *DILI* *concern* according to DILIrank. **c** AEGIS DILI scores from RNA-seq data after treatment of three PHH spheroids donors with compounds with different degree of DILI. Seven compounds are classified as *less* *DILI* *concern* and 12 compounds as *most DILI concern* according to DILIrank. The red area marks a high-risk range of scores, which distinguishes *most DILI concern* from *less DILI concern* compounds with 86% specificity, 75% sensitivity, 90% precision and 0.59 MCC at 10*C_max_. Data presented as the mean DILI score per treatment from 50 iterations. Statistical testing was performed with a two-sided Wilcoxon test. **d** Difference in DILI score (ΔDILI score) for nine pairs of structurally similar compounds, where each pair contains one compound associated with less risk for DILI and the other compound with more risk for DILI. Statistical testing performed with a two-sided one-sample *t*-test. Liver icon from NIAID NIH BIOART (bioart.niaid.nih.gov/bioart/230).

**Fig. 2:**
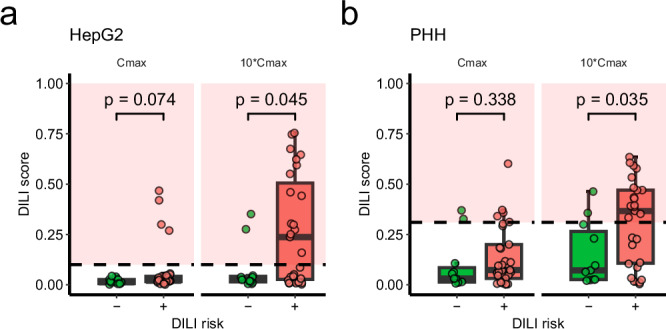
Evaluation of AEGIS on high-throughput transcriptomics data. DILI scores from DRUG-Seq data generated from compound treatment at C_max_ and 10*C_max_ doses in **a** HepG2 cells (*n*** =** 10), **b** two PHH spheroid donors (*n*** =** 5 per donor) after treatment with 11 DILI negative and 29 DILI positive compounds, with DILI annotation based on clinical experience. The red area marks a high-risk range of scores, which distinguishes DILI positive and negative compounds with 89% precision at 10*C_max_. Data presented as mean DILI score per treatment from 50 iterations and statistical testing was performed with a two-sided Wilcoxon test.

An overview of the compounds used in this study are shown in Supplementary Data 1. Figure 1 used DILIrank labels^26^ for the degree of DILI (*less* or *most* *DILI* *concern*. The nine pairs of structurally similar compounds had a Tanimoto similarity score above 0.5^27,28^. Figure 2 used the DILI severity scale^28–30^ to classify compounds as DILI or non-DILI (DILI severity 1–3 for DILI and DILI severity 4–5 for non-DILI).

#### Dosing

**Fig. 3:**
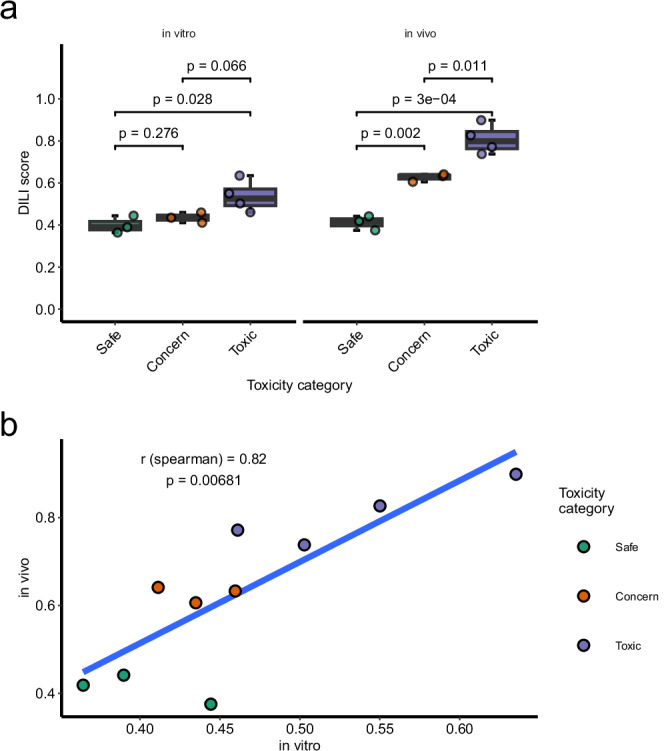
AEGIS prediction of acute Antisense Oligonucleotide toxicity. **a** DILI score from ASO treatment in RAW mouse cell line at 5 µM (*n*** =** 3) and mouse livers from an acute in vivo toxicity study at 150 mg/kg (*n*** =** 3). ASO10 was dosed at 25 mg/kg. ASOs are categorised into toxicity categories: safe, of potential concern, and toxic depending on integrated histopathological scoring, using ALT levels and histopathology findings. Data presented as the mean DILI score per treatment from 50 iterations and statistical testing was performed with a two-sided *t*-test. **b** In vitro to in vivo correlation of DILI scores from ASO treatment.

**Fig. 4:**
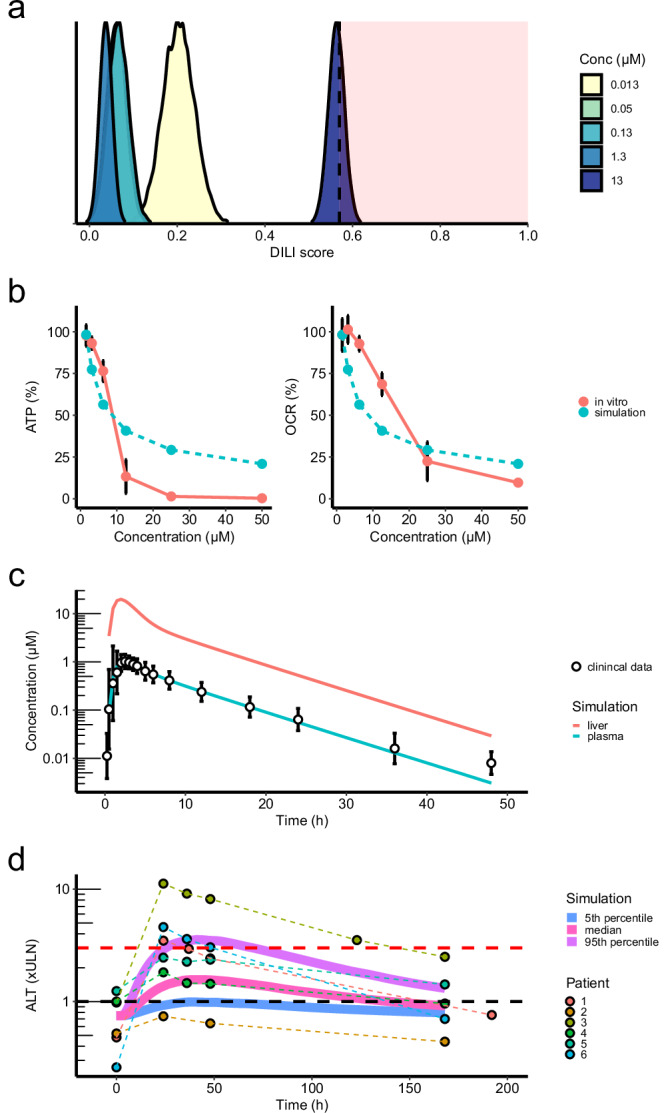
Retrospective safety assessment of AZD1979. **a** Distribution of AEGIS DILI scores after treatment of PHH spheroids from three donors with AZD1979 at concentrations ranging between 0.01 and 10-times C_max_. The red area marks a high-risk zone with 90% confidence that a treatment is at high risk for DILI, see Fig. 1c. **b** Observed and simulated (see Methods for details) change in ATP in galactose media (*n*** =** 3) and basal oxygen consumption rate (OCR) from the Seahorse assay (*n* = 5 for all doses, except *n*** =** 2 for 50 µM and *n*** =** 4 for 1.5625 µM) from HepG2 cells dosed with AZD1979 relative DMSO. Data presented as mean ± standard deviation. **c** PBPK simulation of plasma and liver concentrations of AZD1979 overlaid with clinical plasma levels of AZD1979 from patients receiving 300 mg AZD1979. Clinical plasma levels are visualised as the geometric mean ± geometric mean standard deviation (*n*** =** 6). **d** Dynamic DILIsym® simulation of ALT (normalised to ULN) showing the 5^th^ percentile, median and 95^th^ percentile of 285 individuals from a virtual population incorporating physiological variability of liver function, including ALT turnover and mitochondrial function. Clinical ALT values for six patients receiving 300 mg AZD1979 are shown as circles. Black and red dashed line show 1*ULN and 3*ULN, respectively.

**Fig. 5:**
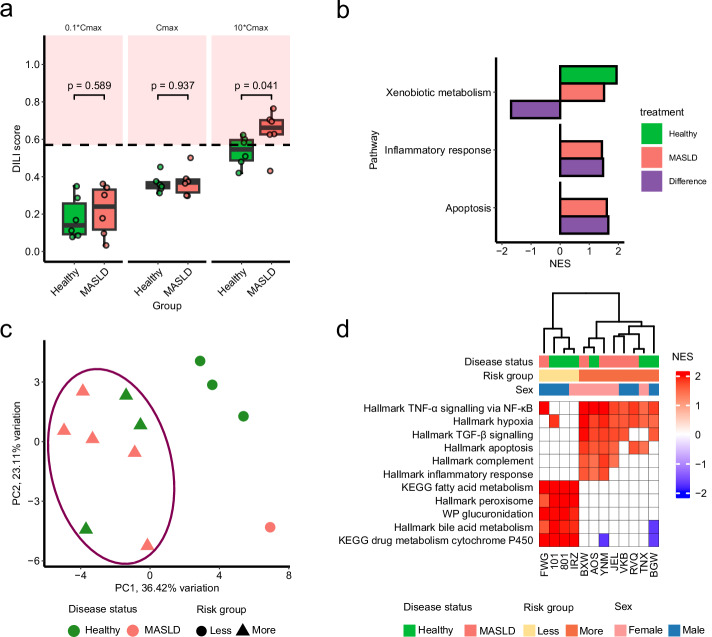
Precision medicine application of AEGIS in the context of MASLD. **a** Donor-specific DILI scores of 2D PHH isolated from healthy and MASLD patients (*n*** =** 6 per group) treated with troglitazone (*n*** =** 3 per donor). Data presented as the mean DILI score per donor from 50 iterations. The red area marks a high-risk zone with 90% confidence that a treatment is at high risk for DILI, see Fig. 1c. **b** Selected gene sets from the Hallmark collection with significant normalised enrichment score (NES) from gene set enrichment analysis of healthy donors and MALSD donors treated with troglitazone at 10*C_max_ compared to vehicle. Full list of significant gene sets in Supplementary Fig. 14. The third column shows the difference in response between the MASLD and healthy. **c** Principal component analysis based on donor-specific hallmark gene set NES from 10*C_max_ troglitazone treatment compared to vehicle. The donors from the high-risk group are inside the high-risk zone from (**a**). **d** Significant NES from 10*C_max_ troglitazone treatment that distinguish the less and more risk groups of donors after evaluating the hallmark, KEGG and WikiPathways (WP) gene set collections. Gene sets with non-significant results (adjusted *p* value >0.05) in (**b**, **d**) are set to 0.

Test compounds were initially solubilised in 100% DMSO prior to dilution in respective experiment dosing media (which contains no FBS) to give 0.2% DMSO (v/v). Compounds were dosed depending on 96/384-well plate format by removing 50/40 µl medium and adding 50/40 µl dosing solution (2x concentration) to give the final test concentration shown in Supplementary Data 1. All small molecule experiments had 7 h treatment period, while 24 h treatment period was used for ASO experiment. Fig. 1 was run as two experiments and used three replicates per donor for DMSO treatment. The DRUG-seq experiment for Fig. 2 included five and eight replicates of compound and DMSO, respectively, per plate. Liver spheroids experiment was conducted with two donors, while HepG2 was conducted with two identical sets of plates. ASO in vitro experiment for Fig. 3 used three and nine replicates of treatment and vehicle, respectively. Fig. 4 used two replicates per donor for DMSO treatment and Fig. 5 used three replicates per treatment per donor.

### Animal experiments for ASOs

An animal study was run according to the AstraZeneca global international standards at an AAALAC International (Association for Assessment and Accreditation of Laboratory Animal Care International)-accredited Contract Research Organisation. About 6–8 weeks old female Balb/c mice (*n* = 3 per group) were randomised based on body weight to minimise confounding effects across groups. Sample size was based on the minimal number of animals possible to allow detection of an acute toxicological finding caused by the test item, hence not powered for statistical analysis. No sex differences in acute tolerability have been observed in earlier studies of ASO, and therefore, only females were chosen to reduce the number of animals needed. All animals were included, no exclusion criteria set. The mice were quarantined for at least 5 days before the study started. They were group housed in polycarbonate cages on a 12-h reverse light-dark cycle with food and water provided ad libitum. The room temperature was 22 ± 3 °C with 40–70% relative humidity. Vehicle (PBS) was sterile filtered. ASOs were administrated as a subcutaneous single dose of 150 mg/kg (dosing volume 15 mg/mL). ASO10 was dosed at 25 mg/kg. Clinical signs and body weights were monitored and recorded daily and after treatment. Animals were euthanized via carbon dioxide at the end of the study 72 h post-dosing. Tissue samples processed for histology (H&E) or put in RNAlater (Ref:AM7021, Thermo Fisher) for RNA-seq overnight at 4 °C before transfer to −80 °C. Blood samples for biochemistry analysis (ALT) were collected at the end of the study and put into tubes without any anticoagulant. The blood samples were centrifuged at 4 °C, 8000×*g* for 15 min before transfer to a new tube and storage at −80 °C.

The severity of DILI from ASOs in mice was categorised by an integrative histopathology scoring approach, using alanine aminotransferase (ALT) levels and histopathology findings. The ALT levels after 72 h in this study ranged from vehicle like <100 U/L to highly toxic (10,000 U/L) and the histopathology findings above vehicle ranged from minimal single cell necrosis to marked degeneration/necrosis. The highest risk category of the two methods were used as the final category. During the study, the group number was known, eg ASO1. During the experimental process and analysis, the group number and animal identification was known. During the data analysis, treatment and group allocation was known.

### Glu-Gal assay mitochondrial toxicity screening assay

HepG2 cells were cultured in 10 mM glucose or 10 mM galactose-containing media (DMEM - no Glucose (11966025, Gibco) and 10% FCS, at 37 °C under humidified air containing 5% CO_2_. For experiments, cells were trypsinised, and seeded at 50,000 cells/well in either glucose- or galactose media in TC-treated 96-well microplates (Sigma) overnight at 37 °C (humidified, 5% CO_2_). Test article was solubilised and serially diluted in DMSO and added to glucose- or galactose media to the required concentrations (0.1% DMSO). The overnight media was removed from cells and replaced by fresh media supplemented with test article (125 µL/well) for 24 h. Cell viability (ATP) was measured by adding an equal volume of 2D CellTiter-Glo® (Promega) assay reagent and incubating at room temperature for 20 min before reading luminescence on an EnVision™ Multiplate Reader. For each concentration of test article, ATP levels were averaged across three technical repeats and normalised to DMSO-treated controls; three independent experiments were performed across three different passages.

### Seahorse assay

The Seahorse XF96 extracellular flux analyzer (Agilent) was used to measure real-time oxygen consumption rates (OCR). HepG2 C3A cells cultured in glucose media were seeded into Seahorse XF96 microplates (Agilent) at a density of 50,000/well and incubated overnight at 37 °C, 5% CO_2_.

The next day, the cells were incubated in unbuffered serum-free DMEM Glucose Seahorse Assay Medium (Agilent) in a 37 °C non-CO_2_ incubator for 1 h. Test article compounds were prepared in the Seahorse Assay medium to the required concentrations and added directly to cells, and cells were immediately loaded into the Seahorse XF96 Analyzer for measurement at 37 °C. The canonical mitochondrial toxins oligomycin A, FCCP and antimycin A were loaded into sensor cartridge injection ports (as described in manufacturers' instructions (Agilent)), and sequentially injected into cells by the Seahorse XF96 extracellular flux analyzer (as described in manufacturers instructions (Agilent)) to yield final concentrations of 1 μM oligomycin A, 1 μM FCCP and 5 μM antimycin A.

Basal OCR was calculated by subtraction of antimycin A-independent OCR from the baseline OCR averaged from the last three measurements prior to oligomycin A addition, and expressed as a percentage of those calculated for DMSO-treated cells; three independent experiments were performed across three different passages.

### RNA isolation and RNA sequencing

For liver spheroid experiments, RNA from ten spheroids/treatment conditions were pooled into a 96-well plate, while a single well was used for the 2D liver experiment. For the ASO experiment, cells were washed twice with PBS (containing Ca+ and Mg+), by centrifugating at 500×*g* for 5 min. Cells were lysed by removing cell culture medium and lysis buffer mix (includes Proteinase K) from the RNAdvance Cell (Beckman Coulter) was added to the cells in a 96-well plate on ice. The cell lysates were thoroughly mixed by pipetting and subsequently incubated at room temperature for 30 min. RNA extraction was performed according to the manufacturer’s protocol on a Biomek i7 Hybrid robotic workstation (Beckman Coulter). Subsequently, mRNA enrichment and the preparation of sequencing libraries were carried out using the KAPA mRNA HyperPrep Kit (Roche) according to the manufacturer’s instructions, using a Tecan Fluent® liquid handler. Library quality was assessed on a fragment analyser using the SS NGS fragment kit (1-6000 bp; Agilent). Sequencing was performed on an Illumina NovaSeq6000 platform (Illumina) at a loading concentration of 1.75 nM.

### RNA-seq data processing

RNA-seq data were processed and quality controlled with an internal Nextflow (v23.10) pipeline. Human genome (GRCh38, Gencode v43) and mouse genome (GRCm39, Gencode vM32) were used. Reads were trimmed by FastP (v0.23.4) and transcript-level counts were calculated with Salmon (v1.10.1) after mapping to a transcriptome index containing cDNA and ncRNA entries, while quality control is based on a STAR (v2.7.11a) alignment. Further quality control was performed using FastQC (v0.12.1), RNAseQC (2.3.5), Samtools (v1.18), and summarised with MultiQC (v1.17).

### DRUG-seq

For liver spheroids, RNA from two spheroids/treatment condition was pooled into a 384-well plate, while a single well was used for HepG2. The DRUG-seq workflow was based on the originally published protocol^31^. Briefly, the compound-containing media were removed, and the treated cells were washed in PBS and lysed at room temperature for 15 min. About 15 uL of cell lysates were transferred into a 384-well V-bottom PCR plate using a TECAN Fluent liquid handling station. Reverse transcription (RT) primers, containing unique molecular identifiers (UMIs), were added to the lysates using an Echo 665 instrument. An RT mix with a Template Switching Oligo (TSO) was then added into each well, followed by incubation at 42 °C for 2 h. After incubation, material from individual wells was pooled and purified using the DNA Clean & Concentrator-100 kit (Zymo Research). Post-exonuclease I (Exo I) treatment, the sample was purified and pre-amplified with DRUG-seq PCR primers. The cDNA pool from every plate was tagmented using loaded Tn5 (Diagenode) and indexed during a final PCR amplification step. The final libraries were then cleaned and size-selected using SPRIselect beads. The quality and quantity of the cDNA and library were assessed using Qubit and the Agilent Fragment Analyzer. Sequencing was performed on a NovaSeq6000, using a loading concentration of 1.85 nM and a custom read primer for Read 1.

### DRUG-seq data processing

The DRUG-seq data were processed with an internal Snakemake (v5.14.0) pipeline based on the original article^31^. Briefly, the reads were trimmed of adaptors with cutadapt (v2.10), aligned to the human genome (GRCh38, Gencode v33) with STAR (v2.7.3a) using STARsolo, demultiplexed, and a UMI count matrix was generated. Quality control was performed with FastQC (v0.11.8), Qualimap (v2.2.2 d), RSeQC (v4.0.0), and summarised with MultiQC (v1.7).

### Microarray TG-GATEs preprocessing

Microarray data from the liver in rat was downloaded from the TG-GATEs^32,33^ public database (https://dbarchive.biosciencedbc.jp/en/open-tggates/desc.html). The CEL files were normalised with robust multi-array average (RMA) normalisation with background correction, using the oligo R-package. Mapping of Affymetrix probes to Ensembl gene identifiers was performed with rat 230_2 (release 36) array annotation. Probes were aggregated to gene-level intensities by selecting the probe with the highest mean intensity across all samples. The rat Ensembl gene identifiers were converted to its human ortholog with the gorth function in the gprofiler2 R-package.

### Transcriptomics analysis

Differential expression analysis was conducted in R with limma^34^. Genes were considered differentially expressed if the Benjamini–Hochberg adjusted *p* value was below 0.05. Gene set enrichment analysis was done with fast gene set enrichment analysis (fgsea)^35^. Input for fgsea was moderated t-statistics from limma. The hallmark gene sets^36^, KEGG pathways and WikiPathways were collected from the Molecular Signatures Database (MSigDB) R-package.

### AEGIS

We selected transcription factors (TF) based on their implications in positive regulation of apoptosis in humans inferred from literature search and GO annotations (Supplementary Table 1). TFs involved with the negative regulation of apoptosis were removed. Furthermore, TFs with only electronic annotation level (IEA) evidence for the positive regulation of apoptosis were removed. From this list, eight TFs were selected based on them being expressed in the liver (TPM >1 in the Genotype-Tissue Expression database): ATF3, E2F3, FOXA1, FOXO3, JUN, PPARG, REST and TFAP4. The gene targets of these TFs were obtained from the non-academic version of Dorothea^37^, with the evidence level A-C. The target genes of the TFs were combined into a gene signature containing 241 genes (Supplementary Data 2).

The AEGIS DILI score was calculated with limma^34^ and the focused gene set testing method ROAST^38^, using the floormean gene set summary statistics and 9999 rotations. The DILI score was defined as the sum of the up and down proportion of activation multiplied by the significance level: (PropUp + PropDown) * −log10(FDR.mixed). Furthermore, the DILI score was min-max normalised to be between 0 and 1 (min = 0, max = 4). The confidence of the DILI scores was assessed by running each treatment 50 times and inserting noise into the expression data based on the gene-level standard deviation across all samples. The mean and standard deviation of the DILI scores from the 50 iterations was used to generate a normal distribution of scores with 10,000 datapoints for visualisation of the AEGIS scores as ridge plots. DILI scores for Figs. 1–5 are shown in Supplementary Data 3.

AEGIS was validated against the Sprague-Dawley rat liver TG-GATEs dataset^32,33^ to link a given DILI score to the fraction of treatments leading to DILI. The in vivo TG-GATEs dataset contains 160 compounds, three doses (1:3:10 concentration ratios for low, middle and high dose) and eight timepoints (3–24 h for single dose and 3–28 days for repeated dose) with timepoint-matched controls for each compound. The compound-dose-timepoint combination leading to DILI was obtained from ref. ^39^ who established nine different disease states based on physiological and histopathological data. The kidney-related disease state (DS9) and disease states without a common transcription profile (DS3 and DS4) were not used. The remaining DILI states (DS1–2 and DS5–8) was used as DILI positives and the non-DS state as DILI negatives. The final list from TG-GATEs had 467 positive and 2684 negative DILI treatments. DILI score for each treatment in TG-GATEs in vivo was calculated by using trend = T in ROAST without noise injection. A stepwise increasing DILI score was used as a cutoff for positive/negative DILI prediction, and the precision was calculated at each DILI score.

### Model metrics

To evaluate the AEGIS performance in binary DILI predictions, we used specificity, sensitivity, precision and Matthew’s correlation coefficient (MCC), computed according to equations 1–4. The MCC metric is optimal for unbalanced datasets. The metrics were calculated as shown below:

1 $${{{\rm{Specificity}}}}=\frac{{{{\rm{TN}}}}}{{{{\rm{TN}}}}+{{{\rm{FP}}}}}$$

2 $${{{\rm{Sensitivity}}}}=\frac{{{{\rm{TP}}}}}{{{{\rm{TP}}}}+{{{\rm{FN}}}}}$$

3 $${{{\rm{Precision}}}}=\frac{{{{\rm{TP}}}}}{{{{\rm{TP}}}}+{{{\rm{FP}}}}}$$

4 $${{{\rm{MCC}}}}=\frac{{{{\rm{TP}}}}* {{{\rm{TN}}}}-{{{\rm{FP}}}}* {{{\rm{FN}}}}}{\sqrt{({{{\rm{TP}}}}+{{{\rm{FP}}}})({{{\rm{TP}}}}+{{{\rm{FN}}}})({{{\rm{TN}}}}+{{{\rm{FP}}}})({{{\rm{TN}}}}+{{{\rm{FN}}}})}}$$

where TP is true positive, TN is true negative, FP is false positive and FN is false negative.

### AZD1979 simulations

Retrospective modelling of AZD1979 clinical hepatotoxicity included physiologically based pharmacokinetic (PBPK) modelling to estimate liver exposure of the drug and quantitative systems toxicology (QST) modelling to simulate the extent of DILI based on the predicted exposure and incorporation of in vitro data on mitochondrial function.

PBPK modelling was performed using GastroPlus®, a software for mechanistic modelling of drug exposure in human tissues. Inputs into the model included drug-specific physiochemical properties and hepatic clearance rate. Model-predicted tissue partition coefficients (Kp) were consistent with results of quantitative whole-body radiography studies in rats, showing a high degree of compound accumulation in the liver (Kp ≈ 10 in in vivo study and model predictions). Clinical plasma data on drug concentration was used to optimise the degree of the observed first-pass effect and drug absorption parameters such that the simulated plasma profile fit the clinical data for 100 and 300 mg dose cohorts, including the cohort in which liver signal was observed (300 mg).

Drug-response profiles resulting from Seahorse assay were used to build an MITOsym®^40^ model of mitochondrial toxicity. Because the assay (and mitochondrial stress test) indicated that the electron transport chain (ETC) is inhibited by AZD1979, the degree of the inhibition in the model was varied to fit the in vitro data. The resulting estimated parameter values and predicted liver exposure were integrated into the DILIsym® model to simulate ALT levels due to mitochondrial toxicity. In order to take into account the population variability of liver physiology, the hepatotoxic effect of the drug was simulated in a virtual population of 285 healthy individuals (SimPops®). The virtual population incorporates (among other parameters) variability of ALT turnover, mitochondrial function, and the corresponding degree of liver injury due to the drug administration. Three sets of DILIsym® simulations were performed corresponding to three cohorts, such that each set of simulations used predicted liver exposure to the respective cohort. Only the simulation of the highest dose cohort resulted in ALT elevations.

### Statistics and bioinformatics

Two-sided Student’s *t*-test was used when data followed normality (tested with qqplot, Shapiro test and Levene test), otherwise two-sided Wilcoxon test. R (v4.1.0) was used for analysis and visualisations of the results in the paper. The following R-packages were used: ComplexHeatmap (v2.14.0), data.table (v1.14.0), drc (v3.0-1), edgeR (v3.36.0), fgsea (v1.20.0), ggpubr (v0.6.0), ggridges (v0.5.6), gprofiler2 (v0.2.0), limma (v3.50.3), msigdbr (v7.5.1), oligo (v1.55.1), parallel (v4.1.0), patchwork (v1.1.2), PCAtools (v2.6.0), plyr (v1.8.8), reshape2 (v1.4.4), rstatix (v0.7.2), tidyverse (v2.0.0) and yardstick (v1.2.0).

## Results

### AEGIS accurately predicts dose-dependent DILI in the rat liver gene expression dataset

We chose apoptosis as a universal marker of DILI, as the mechanisms of toxicity for many DILI drugs converge on this process^21,41^, and selected eight transcription factors (see Methods) that positively regulate apoptosis and are expressed in the liver (Supplementary Table 1 and Fig. 1a). Their target genes were combined into a set of 241 genes, with the transcription factors JUN being the biggest contributors to the set with 53 genes (Supplementary Fig. 1a). Confirming our focus on perturbation of apoptosis-regulating pathways, over-representation analysis against the Gene Ontology Biological Processes sets revealed significant enrichment for processes involving xenobiotic stimulus, oxidative stress and apoptosis signalling pathways in the AEGIS signature (Supplementary Fig. 1b). The AEGIS algorithm can then be applied to gene expression data from a comparison between treatment and control to calculate a DILI score (see Methods). This DILI score was evaluated against the TG-GATES^32^ rat liver gene expression dataset containing 160 compounds across three doses (see Methods), with negative and positive DILI labels per treatment (‘treatment’ meaning a compound-dose-timepoint combination) in rat^39^. The DILI score captured the dose response in TG-GATEs (log-logistic model, Hill slope = −0.69 and EC50 = 56.4, *p* < 2.2e-16 for both; Supplementary Fig. 2). DILI-positive treatments in TG-GATEs resulted in significantly higher DILI scores than DILI-negative treatments (p < 2.2e-16, two-sided Wilcoxon test; Supplementary Fig. 3a). The best performance of the DILI scores to predict DILI was obtained at a DILI score threshold of 0.36, achieving a Matthew’s correlation coefficient (MCC) of 0.39. Furthermore, increasing DILI score was correlated to increasing DILI prediction precision (fewer false positives), indicating that the confidence of the DILI score to predict DILI improves with increasing DILI score (Supplementary Fig. 3b). High precision and specificity are desirable in early testing to avoid incorrect stopping of safe compounds. Looking at perturbation of the regulons of AEGIS transcription factors individually, ATF3 had most frequently the highest perturbation (Supplementary Fig. 4).

This validation of AEGIS against the rat liver TG-GATEs dataset demonstrated the ability of the AEGIS DILI score to identify DILI treatments in rats, with increasing DILI score corresponding to increasing confidence in the DILI predictions.

### AEGIS distinguishes compounds with different degrees of DILI concern and compound pairs with high structural similarity based on clinical outcomes

Our initial validation showed that increasing AEGIS DILI score correlates with increasing confidence that a treatment is at risk of causing DILI, based on a specific degree of apoptotic perturbation in rats. This degree of perturbation is dependent on the number of differentially expressed (DE) genes within the AEGIS signature set, which in turn is dependent on the species and the technology used to generate transcriptomics data. To establish the translatability of DILI scores generated from in vitro models to human clinical outcomes, we treated primary human hepatocyte (PHH) spheroids from three different donors with small molecule compounds of varying DILI risk, using compound DILI annotation data from DILIrank^26^. This classification of clinical drugs is based on FDA-approved drug labelling documents and literature evidence^26^, and groups compounds into the categories *no*, *less*, *ambiguous* and *most DILI concern*. During preclinical drug discovery, efficacious compounds that are evaluated might display a range of DILI risks, with the lowest risk compound prioritised for development. We selected 22 compounds with described clinical DILI outcome (7 *less*, 3 *ambiguous*, and 12 *most* *DILI* *concern*, Supplementary Data 1) to evaluate AEGIS’ ability to rank DILI compounds based on their degree of DILI concern, at clinically relevant doses. PHH spheroids were dosed at 0.1-, 1- and 10-times clinical C_max_ for 7 h before transcriptional profiling for AEGIS using RNA-seq. The 7 h treatment time was chosen to assess the acute genetic signature and to avoid a general cytotoxic effect.

AEGIS captured the dose response of the compounds (log-logistic model, Hill slope = −0.48 and EC50 = 9.71, *p* value of 2.92e-09 and 2.06e-03, respectively; Supplementary Fig. 5). An example output of the DILI scores of zolpidem and alpidem treatment are shown in Fig. 1b, where alpidem is labelled *most* *DILI* *concern* and zolpidem *ambiguous DILI* *concern* in DILIrank^26^. AEGIS predicts that alpidem has a stronger apoptotic response and thus a greater chance of causing DILI at C_max_ and 10*C_max_ compared to zolpidem, consistent with the DILIrank annotation. Despite preclinical and clinical safety testing, alpidem was withdrawn from the market in 1995, while in vitro analysis using AEGIS would have highlighted the risk it posed to patients.

Classification of the seven *less* and 12 *most DILI concern* compounds with AEGIS at 10*C_max_ resulted in prediction metrics of 86% specificity, 75% sensitivity, 90% precision and 0.59 MCC, using a DILI score threshold of 0.57, modified for RNA-seq in human because of increased power to detect DE genes (Fig. 1c). This threshold defines a high-risk zone for DILI that can assist the decision-making process during drug discovery, ensuring that safe compounds are not incorrectly stopped. If a candidate compound reaches this zone at a given dose, we can be 90% confident that this dose is at high risk for DILI, and the therapeutic margin of the compound should be established before considering stopping the compound from further progression. AEGIS produced an accurate DILI classification at 10*C_max_ (two-sided Fisher’s exact test: *p* = 0.02, 95% CI: 1.15–910.1, OR: 14.9), while compounds which proved challenging for AEGIS, such as cyclofenil, may be explained by the low transcriptomics response to these compounds, resulting in the lack of significance in scores between groups (*p* = 0.12, two-sided Wilcoxon test). Comparing less* DILI* and most* DILI* compounds without cyclofenil showed a significant difference in AEGIS score between the groups (*p* = 0.044, two-sided Wilcoxon test).

A series of lead compounds might have high structural similarity, meaning phenotypic assays with a limited set of endpoints may have difficulty discriminating between compounds in a series. While this also presents a challenge to compound prioritisation using transcriptomics, transcriptome-wide data may provide greater resolution and an opportunity to improve over phenotypic assays, using a transcriptomics-based DILI predictor such as AEGIS. This challenge, therefore, presents an opportunity to provide insights about compound tolerability beyond what structural analyses could provide. We therefore investigated if AEGIS could rank structurally similar compounds based on their safety profile. In the previously generated data, we had nine pairs of compounds with high Tanimoto similarity ( > 0.5). In each pair, one compound was associated with less liver injury (*less* or *ambiguous DILI concern*) and the other with more liver injury (*most DILI concern*). This allowed us to investigate if AEGIS can distinguish the more toxic drug from its less toxic structural analogue in each pair. At 10*C_max_, AEGIS assigned a higher DILI score to the more toxic compounds in the pairs (*p* = 0.033, two-sided one-sample *t*-test), with a median increase of 38% in DILI score (Fig. 1d). While six pairs were correctly ranked, three pairs of structural analogues were either wrongly ranked or not separated: ibuprofen-ibufenac, ethacrynic acid-ticrynafen and clomiphene-cyclofenil (Supplementary Fig. 6). Ibuprofen had higher scores than ibufenac, ticrynafen had a higher DILI score at 0.1*C_max_ and C_max_ but overlaps with ethacrynic acid at 10*C_max_, and both clomiphene and cyclofenil had a low DILI score.

Taken together, these data demonstrate that AEGIS allows us to help prioritise compounds based on clinical DILI concern during drug discovery, including when evaluating structurally similar compounds, which is critical in guiding the design of compounds to minimise risk before clinical trials.

### AEGIS accurately separates negative/positive DILI labels using low-depth transcriptomics technology

Deep RNA-seq is the gold standard transcriptomics technology, but can be too expensive for high-throughput screening of many compounds during drug discovery. Therefore, we investigated if AEGIS could be applied to transcriptomics data generated from a different platform: shallow global RNA-seq with DRUG-seq^31^.

DRUG-seq quantifies gene expression by sequencing only the 3’ end of transcripts, thus generates data at a lower cost and depth compared to standard RNA-seq. We treated HepG2 cells and PHH spheroids from two different donors for 7 h with 11 negative and 29 positive DILI compounds according to the Garside DILI labelling^28–30^ of clinical drugs (Supplementary Data 1). Garside provides a DILI severity category between 1 and 5, where categories 1–3 was defined as DILI positive and 4–5 as DILI negative. Compounds were dosed based on their clinical C_max_ values. Both HepG2 and PHH spheroids obtained higher DILI score for DILI positive compounds compared to DILI negative compounds when dosed at 10*C_max_ (*p* = 0.045 and 0.035, respectively, two-sided Wilcoxon test; Fig. 2a, b). Furthermore, classifying compounds based on AEGIS predictions at 10*C_max_ for both HepG2 and PHH spheroids resulted in 82% specificity and 89% precision (DILI score threshold of 0.1 and 0.31, respectively). The use of PHH instead of HepG2 increased the sensitivity from 55 to 59% and the MCC from 0.33 to 0.36. AEGIS DILI scores from DRUG-seq were overall lower compared to RNA-seq (Figs. 2a, b, 1c), possibly due to the lower numbers of differentially expressed genes detected by the shallower DRUG-seq (Supplementary Fig. 7).

The DRUG-seq dataset also included 7 of the 9 Tanimoto similar pairs from the RNA-seq dataset (Supplementary Fig. 8a). In the PHH dataset, AEGIS assigned a higher DILI score to the more toxic compound in the pairs at 10*C_max_ (*p* = 0.0142, two-sided one-sample *t*-test; Supplementary Fig. 8b), with a 54% median increase in DILI score. The only pair not separated was clomiphene-cyclofenil, both with weak transcriptomic responses. The DILI scores of the pair ethacrynic acid-ticrynafen was not distinguishable in RNA-seq data (Supplementary Fig. 6) but now correctly ranked in DRUG-seq data (Supplementary Fig. 8a).

Together, these findings demonstrate that AEGIS can accurately distinguish the DILI signatures from positive and negative compounds in a low-depth transcriptomics technology and across in vitro models. Combining AEGIS with a high-throughput and low-cost transcriptomics technology could possibly accelerate drug discovery by allowing more compounds to be evaluated.

### AEGIS predictions of safety liabilities in oligo-based therapies translate to histopathological findings

Having demonstrated the ability of AEGIS to detect a range of liver injury induced by small molecule therapies, we asked whether the approach is also applicable to other therapeutic modalities. Antisense oligonucleotides (ASOs) are a class of nucleotide-based therapeutics that act via Watson-Crick base pairing. This allows for treating both rare and common diseases by several different mechanisms, including reducing the abundance of a specific protein through degradation of its transcript or by modulating incorrect splicing of pre-mRNA^42^. Some cases of liver and kidney toxicity have led to withdrawal of FDA-approved ASO compounds from the market^43^. Early identification of these risks could assist in prioritising progression of the safest ASOs, reduce attrition, and accelerate drug discovery. Also, different properties and mechanisms of action of ASOs make assays used for the evaluation of small molecules less applicable to the prediction of ASO hepatotoxicity^44^. Therefore, we investigated whether AEGIS could detect hepatotoxicity from ASOs and assessed in vitro to in vivo translation by evaluating transcriptomic profiles of 10 different ASOs with different liver injury potential in both the RAW 264.7 mouse cell line and a 3-day acute study in Balb/c mice subcutaneously administered with high doses of ASOs (150 mg/kg).

The in vivo toxic response from ASOs, based on a combination of integrated histopathological scoring (from minimal single cell necrosis to marked degeneration/necrosis) and in vivo alanine aminotransferase (ALT) levels (up to >10,000 U/L), were classified into three groups of liver injury: safe, of potential concern, and toxic. ASOs categorised as toxic obtained significantly higher in vitro DILI scores from AEGIS compared to ASOs categorised as safe (mean ΔDILI score = 0.14, 95% CI: 0.02–0.25, *p* = 0.028, two-sided *t*-test; Fig. 3a). There was no significant difference between ASOs categorised as of potential concern compared to safe or between potential concern and toxic in vitro. In mouse liver, AEGIS DILI scores from ASO treatment were significantly higher in both the potential concern and toxic category compared to safe, with mean ΔDILI score of 0.21 (95% CI: 0.15–0.28, *p* = 0.002, two-sided *t*-test) and 0.40 (95% CI: 0.29–0.50, *p* = 0.000326, two-sided *t*-test), respectively (Fig. 3a). Additionally, there was a significant difference between toxic and potential concern (mean ΔDILI score of 0.18, 95% CI: 0.07–0.29, *p* = 0.011, two-sided t-test). To evaluate the accuracy of AEGIS between in vitro and in vivo models, we compared the correlation between their DILI scores. The ASO in vitro scores showed a strong positive relationship with the in vivo scores (Spearman correlation = 0.82, *p* = 0.00681; Fig. 3b). Reducing animal use is a key factor during drug development, and these data indicate good translatability between in vitro and in vivo predictions.

Together, these data show that AEGIS scores are not only informative when applied to small molecules but can be applied to data from other drug modalities, such as ASOs, to identify safety concerns and prioritise which compounds to progress into in vivo studies.

### AEGIS predictions of safety liabilities during the drug development of small molecules translate to findings from clinical trials

The development of new assays and prediction tools could allow earlier detection of safety concerns of compounds during drug discovery. Early identification of safety signals can guide the prioritisation and optimisation in a chemical series or, if necessary, stop the compound before reaching the clinic. AZD1979 is a melanin-concentrating hormone receptor 1 (MCHR1) antagonist that was developed as a treatment for obesity^45^. AZD1979 showed no signs of DILI risk in animal toxicity studies, but ALT increases observed during Phase 1 clinical studies led to project discontinuation^1^. Out of six patients administered a 300 mg dose, five exceeded the upper limit of normal (ULN) for ALT levels, and three exceeded 3*ULN. As a case study within the context of drug development in the pharmaceutical industry, we evaluated the ability of AEGIS to impact AstraZeneca’s drug development pipeline by applying the method retrospectively to AZD1979.

We conducted retrospective in vitro hepatotoxicity analysis of AZD1979 in PHH spheroids from three individual donors and generated RNA-seq for AEGIS assessment. AZD1979’s effect yielded DILI scores inside the high-risk zone at 10*C_max_ (Fig. 4a). Pathway analysis revealed significant enrichment for downregulated genes within the oxidative phosphorylation pathway at C_max_ and 10*C_max_ (Supplementary Fig. 9). In agreement, AZD1979 exhibited increased toxicity in the galactose-arm versus the glucose-arm of the HepG2 mitochondrial toxicity Glucose/Galactose screening assay (Supplementary Fig. 10a). Real-time oxygen consumption rates (OCR) measurements using Seahorse assays in HepG2 cells confirmed that AZD1979 rapidly inhibits mitochondrial electron transport chain activity at clinically relevant concentrations (Supplementary Fig. 10b). The changes in ATP in galactose media and OCR due to the AZD1979-induced inhibition were modelled using MITOsym®^40^, a mathematical model of hepatocellular respiration and bioenergetics (Fig. 4b). Physiologically based pharmacokinetic (PBPK) modelling showed high exposure of AZD1979 in the liver, with liver C_max_ reaching 19 times plasma C_max_ for patients receiving 300 mg AZD1979 (Fig. 4c). Integrating liver exposure predictions and MITOsym® simulation outputs into DILIsym®^46^, a quantitative systems toxicology (QST) model, predicted ALT elevation patterns consistent with clinical findings (Fig. 4d). Modelling liver exposure and mitochondrial toxicity at 100 mg AZD1979 did not predict ALT elevations, also in line with clinical observations (Supplementary Fig. 11).

In retrospect, if AEGIS had been available at the time, it is possible that the safety concerns associated with AZD1979 would have been highlighted, allowing them to be mitigated before progressing into clinical trials.

### AEGIS captures increased DILI severity from Troglitazone treatment in hepatocytes isolated from MASLD patients

Risk of DILI may vary depending on a number of susceptibility factors, being genetic or nongenetic^47^, with pre-existing liver conditions a suspected risk factor^16^. Precision medicine could allow mitigation of DILI incidences by identifying at-risk patient groups and tailoring treatments accordingly. Therefore, we applied AEGIS in a precision medicine context to evaluate its capability to detect differences in DILI risk based on underlying liver disease, using metabolic dysfunction-associated steatotic liver disease (MASLD) as proof-of-concept. MASLD has caused pharmacokinetic changes in the liver of patients^20^ and is suspected to increase the risk for DILI^48,49^. By applying AEGIS on public liver biopsy RNA-seq data from MASLD patients^50,51^, we showed that increasing MASLD severity correlates with higher AEGIS DILI scores (Supplementary Fig. 12). A compound with suspected risk of increased DILI severity in MASLD patients is troglitazone^52^. Troglitazone has been shown to increase toxicity in a MASLD model of HepaRG cells^22^, organoids^23^ and rats^24^, although the effect of MASLD on increased clinical DILI risk remains unclear. Troglitazone is a PPAR-γ agonist that was used to enhance insulin sensitivity in patients with type 2 diabetes but was withdrawn from the market in 2000 by the FDA due to numerous cases of liver failure from DILI.

We investigated whether AEGIS could differentiate DILI severity from troglitazone treatment in healthy versus MASLD patients. Isolated 2D PHHs from six donors (three males and three females) from each group were treated with troglitazone. Cells from patients with mid-stage MASLD progression (NAFLD activity score of 3; sum of the degree of steatosis, ballooning and lobular inflammation) were chosen because more advanced MASLD involves a complex combination of fibrosis^53^ and inflammation^54^, which might not be retained in isolated hepatocytes. The disease phenotype of the isolated hepatocytes from MASLD donors was verified by comparing gene expression profiles between the vehicle treatments of the MASLD group and the healthy group. Gene set enrichment analysis revealed significant enrichment for upregulated genes in the MASLD group for innate immune response, unfolded protein response and mTORC1 signalling (Supplementary Fig. 13a), which are implicated in MASLD pathogenesis^54–56^. Furthermore, known NAFLD/NASH signatures^57,58^ were significantly enriched in the MASLD group (Supplementary Fig. 13b). MASLD donors also exhibited significant up-regulation of *CYP2E1* (log_2_fold change = 0.58), which have been observed in MASLD patients and might increase the risk for acetaminophen hepatotoxicity^59^.

Treatment of isolated PHH with troglitazone at 10*C_max_ resulted in significantly higher AEGIS DILI scores in MASLD donors compared to the healthy donors (p = 0.041, two-sided Wilcoxon test; Fig. 5a). All MASLD donors, except one, had DILI scores in the high-risk zone, whereas only half of the healthy donors did. This stronger apoptotic response in the MASLD group indicates that these patients may develop more severe DILI from troglitazone. Both groups showed significant positive enrichment of xenobiotic metabolism, but this induction was significantly lower in MASLD donors (Fig. 5b and Supplementary Fig. 14). Moreover, only MASLD donors had a significant positive enrichment of apoptosis and inflammatory response. Enhanced apoptotic and inflammatory responses are markers of DILI^60^ and indicate more severe DILI in MASLD donors.

Investigating the transcriptomic response at 10*C_max_ in individual donors identified two risk groups: less (three healthy, one MASLD) and more (three healthy, five MASLD) (Fig. 5c and Supplementary Fig. 15a, b). These groups align with the obtained DILI scores, as the donors in the less risk group were below the high-risk zone. Distinct transcriptional response of the *less* risk group was significant positive enrichment of cytochrome P450 pathways, fatty acid metabolism, bile acid metabolism, peroxisome, and glucuronidation (Fig. 5d). Calculating the perturbation of individual transcription factors in the AEGIS gene signature revealed that donors in the less risk group have lower perturbation of the transcription factors FOXA1 and JUN (Supplementary Fig. 16). Features of the *more* risk group included positive activation of TNF-α signalling via NF-κB, hypoxia, TGF-β signalling, apoptosis, and immune response pathways (Fig. 5d).

We demonstrated that AEGIS could detect differences in DILI score from troglitazone treatment based on underlying liver disease. This indicates that AEGIS can be used to identify drugs that may be more harmful in specific subgroups of patients.

## Discussion

Early detection of safety concerns during drug discovery is important for their mitigation, or to prevent progression to clinical trials, which could present a risk to patients. We have developed AEGIS: a tool to estimate risk for intrinsic DILI from transcriptomics data generated preclinically. AEGIS calculates a DILI score that corresponds to the perturbation of apoptosis-regulating transcription factors. In this study, we show that AEGIS can be used to rank compounds during drug discovery across different species, transcriptomics technologies, in vitro models and modalities.

With AEGIS, we can provide a quantitative risk score of DILI from transcriptomics data, which is lacking in conventional risk assessment with pathway analysis. This quantitative risk score can guide projects during compound selection and design to further improve safety. The main use of AEGIS is not binary DILI predictions, but for evaluating dose responses and ranking of compounds in terms of safety. Still, a threshold for toxic compounds can be informative to drug discovery efforts. Therefore, we established a high-risk zone for RNA-seq data that distinguishes *less* and *most* *DILI* *concern* compounds with 86% specificity, 75% sensitivity and 90% precision (Fig. 1c). These metrics are comparable to binary DILI predictions obtained from bioactivity assay endpoints in PHH spheroids^28,61^, human liver microtissues^30^ and liver chips^62,63^. Reflecting the basis of AEGIS in measuring transcriptional perturbation of a focused gene set, is the lack of detection of DILI compounds with a low transcriptomics response (e.g. cyclofenil). Addressing this requires testing at different onset times, integration of various omics data, and/or developing advanced organoid models from patient-derived cells. Furthermore, future expansion of AEGIS involves providing DILI mechanistic insights by generating quantitative risk scores per DILI mechanism. Understanding which DILI phenotype is responsible for hepatotoxicity is important for drug discovery projects to redesign compounds and mitigate DILI risks.

AEGIS is compatible with multiple transcriptomics technologies, and we demonstrated accurate DILI predictions with AEGIS from microarray, RNA-seq and DRUG-seq data. AEGIS with DRUG-seq data was also able to separate structurally similar compounds based on their risk of DILI (Supplementary Fig. 8). Use of AEGIS and DRUG-seq shows great promise during early drug discovery when low-cost, high-throughput screening of compounds is needed. The drawback is the drop in sensitivity between AEGIS predictions on RNA-seq and DRUG-seq data (from 75 to 59%; Figs. 1c and 2b). Improved predictivity on DRUG-seq data could be achieved by adjusting the threshold after testing more compounds and by optimising the DRUG-seq method. This flexibility of AEGIS means that it can be implemented either for screening in a high-throughput manner (e.g. DRUG-seq) or, if deep investigation of the mechanism of action is also required, with the deeper sequencing technology RNA-seq. Switching from DRUG-seq to RNA-seq data in later stages of drug discovery might be necessary when the list of candidate compounds is shorter, and a higher prediction accuracy is needed.

An alternative method for high-throughput safety assessment was proposed by Kang et al. with the use of qPCR^14^. Their approach achieves similar predictability as AEGIS, but they are only assessing the expression of ten genes, which limits further mechanistic analysis of the DILI phenotype, enabled by the generation of transcriptome-wide data used by AEGIS. Stolte et al. demonstrated that cytotoxicity and gene expression data can be combined for high-throughput prediction of safety, with similar metrics as AEGIS^15^. However, their approach was only tested on targeted RNA-seq data (TempO-Seq), and the use of an XGBoost model (an implementation of gradient boosted decision trees) could result in limited interpretability of the model for further safety investigations.

By basing AEGIS on fundamental biology, we could achieve a drug-modality agnostic safety assessment. Within the oligo-based therapy modality, AEGIS predicted a higher DILI score for ASOs that was consistent with the histopathological findings. The current de-risking strategies for ASOs rely heavily on animal testing^64^, with most conventional in vitro cellular models not suitable for ASO safety screening^44^. We have shown that AEGIS can be used as a tool to select which ASOs to progress to in vivo testing. While the in vitro predictions might be improved by using an organoid model instead of a macrophage cell line, there was still good translatability between the in vitro and in vivo DILI scores (Fig. 3b). However, a low number of animals per group was used (3R considerations), which could limit the final evaluation due to interindividual variation. Verifying this translatability by testing more ASOs could pave the way towards reducing animal testing.

A particularly powerful finding from this study was that AEGIS detected risk of DILI for AZD1979, a compound that did not show safety concerns in animal models but was stopped during clinical trials due to ALT elevations^1^. This example highlights the strength of incorporating transcriptomics data into safety assessment. In the case of AZD1979, using AEGIS would have flagged a DILI risk. Further pathway analysis utilising the transcriptomics data would have revealed pathways involved with mitochondrial toxicity, prompting follow-up safety assessment of AZD1979. Furthermore, additional safety liabilities were uncovered from the quantitative systems modelling (integrating seahorse assay data and PBPK modelling), which predicted clinically significant ALT elevations from high liver exposures. The insights from the high AEGIS score, the biochemical signals, and the QST/PBPK modelling can be used to mitigate the safety concerns by steering away from certain types of chemistry when improving the compound. These insights, which are informing safety screening approaches at AstraZeneca, also demonstrate the strength of combining different models, which resulted in accurate translatability to clinical outcomes.

In the age of personalised medicine, it is crucial that the possibility of variations in response to treatment are incorporated from an early stage. The highly prevalent disease MASLD might increase the risk for DILI from certain drugs^48,49^. Clinical observation suggests a fourfold increase in DILI incidence among patients with MASLD^65,66^ (from 0.2 to 0.8%^66^), with an incidence rate ratio of 4.42 after MASLD diagnosis^67^. Another study found a threefold higher mortality (16 vs 5.2%) for patients with underlying liver disease^68^. We demonstrated that troglitazone treatment of cells from patients with MASLD received a higher DILI score than cells from healthy patients. This indicates a higher severity of DILI in MASLD patients, which aligns with the mentioned clinical observations. Constructing MASLD organoid models^69,70^ and including more donors could improve the AEGIS predictions further. However, this hypothesis needs to be validated clinically following the Roussel Uclaf Causality Assessment Method (RUCAM)^71^ to determine clinical causality. Using AEGIS in precision medicine could assist in patient stratification and identify patient groups that are at higher risk of developing DILI from a specific drug. These patient groups could either be recommended a reduced dose or an alternative drug.

This work focuses on intrinsic DILI, a leading cause of drug attrition in early drug discovery. AEGIS was developed and validated on retrospective datasets spanning both failed and safe drugs, and its prospective performance will be closely monitored as it is deployed in future drug discovery programs alongside additional risk assessment methods. Importantly, AEGIS is not intended to address idiosyncratic DILI, which often involves an immune-mediated component^72^ that is not captured in isolated hepatocyte systems. Thus, a low-risk DILI score does not exclude immune-mediated DILI. Moving forward, assessing DILI risk in patients with immunological susceptibilities will require improving the AEGIS model and pairing it with advanced in vitro models, such as organoids and co-cultures, to better model immune-driven mechanisms of DILI.

With this study, we have demonstrated the versatility of our DILI risk assessment tool AEGIS. AEGIS can be successfully applied to different species, in vitro models, sequencing technologies, and modalities. AEGIS provides a quantitative risk score that can be used to prioritise compounds in terms of safety, with the goal of providing safer medicines to patients.

## Supplementary information

Supplementary Information

Description of Additional Supplementary files

Supplementary Data 1

Supplementary Data 2

Supplementary Data 3

Supplementary Code

## Data Availability

The AEGIS gene signature, containing 241 genes, can be found in Supplementary Data 2. The gene counts and metadata for Figs. 1, 2, 4, 5 are available on ArrayExpress under the accession numbers E-MTAB-15586 (Fig. 1, experiment 1), E-MTAB-15587 (Fig. 1, experiment 2), E-MTAB-15601 (Fig. 2, HepG2), E-MTAB-15612 (Fig. 2, PHH), E-MTAB-15609 (Fig. 4), E-MTAB-15610 (Fig. 5). The raw RNA-sequencing data from human samples cannot be shared due to Swedish law, patient consent, and the risk of identifying the patients from the sequencing data. Additional information and data for ASOs are proprietary and cannot be shared. The source data for Figs. 1–5 can be found in Supplementary Data 3.

## Code availability

Custom R code for calculating the AEGIS DILI scores is available as a supplementary file (AEGIS_v.1.0_example.R).
