# Supplementary material for: Apoptotic signatures allow early and rapid screening of drug-induced liver injury to accelerate drug discovery
Source: Commun Med (Lond). 2025 Dec 24;6:48. doi: 10.1038/s43856-025-01306-7 (PMC12827481; doi:10.1038/s43856-025-01306-7)
Supplement: Supplementary file 1 — Supplementary Information [file 43856_2025_1306_MOESM1_ESM.pdf]

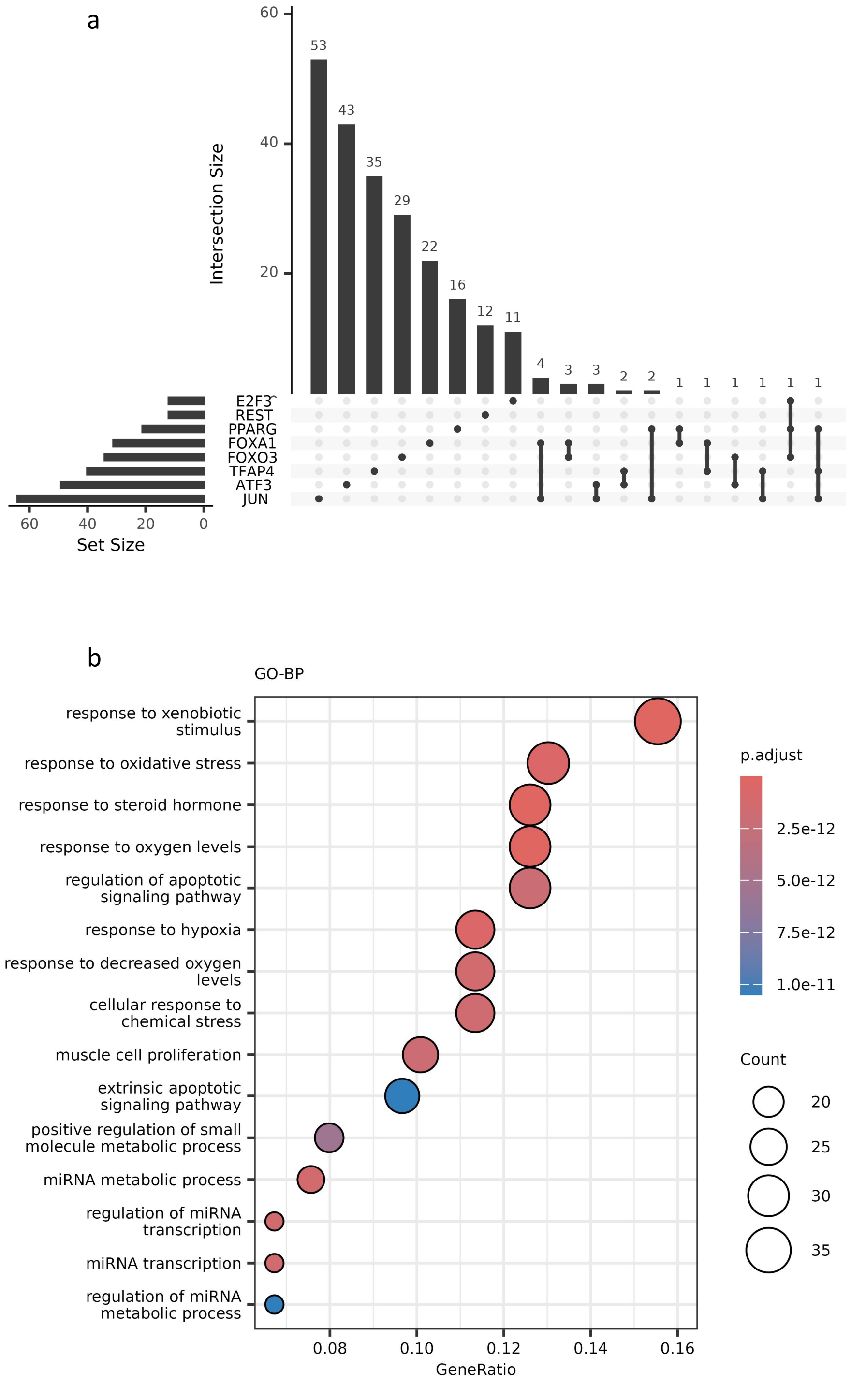

Figure 1. Overview of AEGIS regulons. (**a**) UpSet visualization of gene target overlaps between the eight transcription factors in the AEGIS gene signature. (**b**) Over-representation analysis of the AEGIS gene signature, using the Gene Ontology Biological Processes sets.

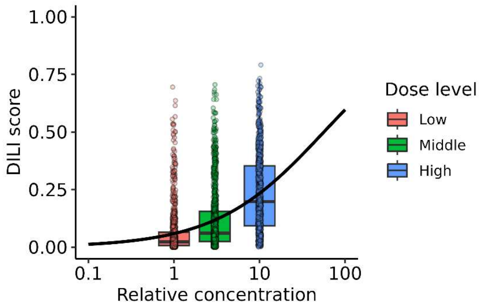

Figure 2. Dose response of AEGIS DILI score on microarray data from the TG-GATEs *in vivo* dataset. Dose response function was fitted with the R-package drc as log-logistic model on the ratio between the doses low, middle, high as 1:3:10. Resulting model had Hill slope = -0.69 and EC50 = 56.4 with $p < 2.2e-16$ for both parameters.

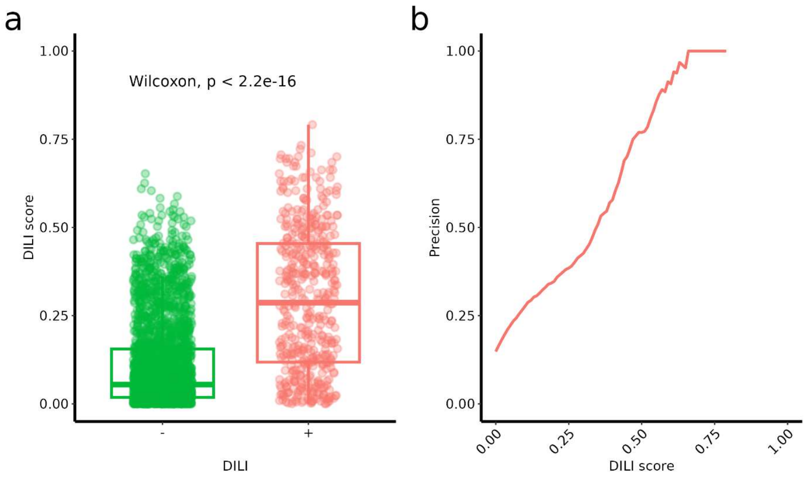

Figure 3. AEGIS performance on microarray data from the TG-GATEs rat liver dataset. (**a**) DILI score for each compound-dose-timepoint combination that was identified as either DILI negative (n = 2684) or positive (n = 467) by Shimada et al. (2019). (**b**) Change in precision with increasing DILI score as threshold for DILI classification.

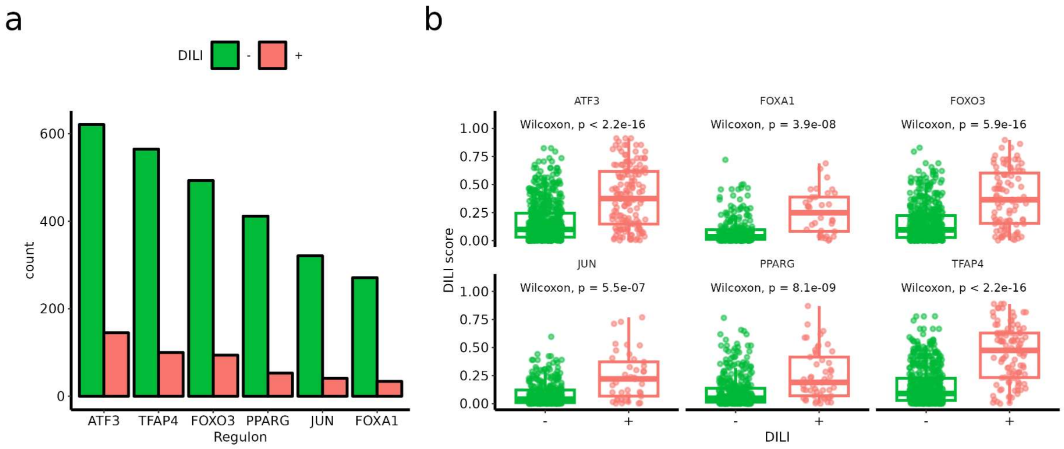

Figure 4. Highest scoring AEGIS regulon in the microarray data from TG-GATEs rat liver dataset. (**a**) Frequency of AEGIS regulon that obtain the highest score in the treatments (compound-dose-timepoint) of TG-GATEs. (**b**) Differences in DILI score of the highest scoring regulon between DILI negative and DILI positive treatments (labelled according to Shimada et al. (2019)). Only AEGIS regulons with more than 10 genes present in the dataset were evaluated.

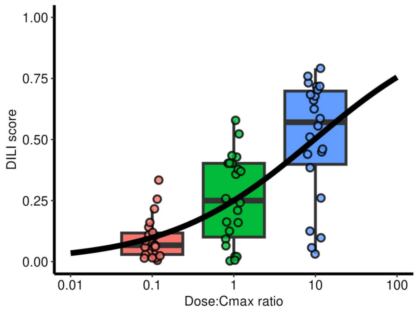

Figure 5. Dose response of AEGIS DILI scores on RNA-seq data from primary human hepatocyte spheroids. 22 compounds (7 less, 3 ambiguous and 12 most DILI concern from DILIrank) were dosed at 0.1-, 1- and 10-times C_{max}. Dose response function was fitted with the R-package drc as log-logistic model and resulting model had Hill slope = -0.48 and EC50 = 9.71 with p-value of 3.92e-09 and 2.06e-03, respectively.

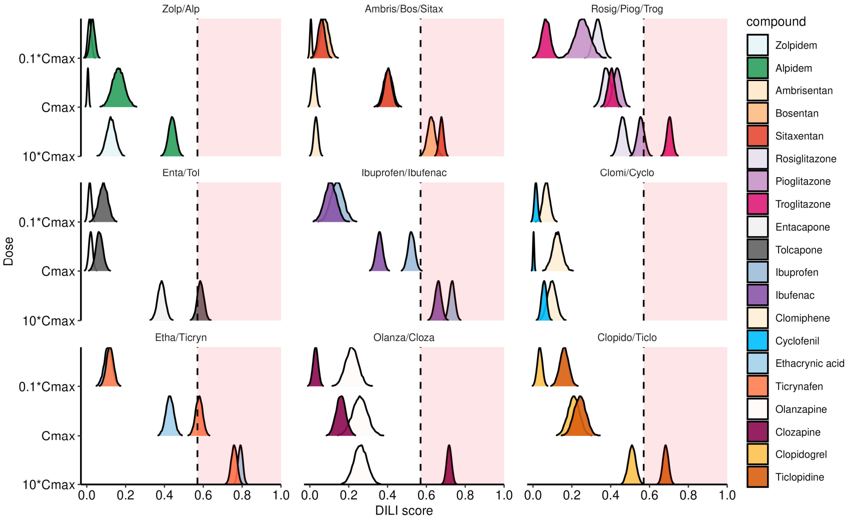

Figure 6. Distribution of AEGIS DILI scores from 9 pairs of Tanimoto similar compound. Compounds were dosed at 0.1-, 1- and 10-times C_{max} in primary human hepatocyte spheroids and AEGIS DILI score calculated from RNA-seq data. Darker colour corresponds to increasing Dose-Based DILI score from Chen et al. (2016) within a pair. The distributions are generated by running AEGIS 50 times and inserting noise into the expression data based on gene-level standard deviation across all samples. The red area marks a high-risk zone with 90% confidence that a treatment is at high risk for DILI, see **Fig. 1c**.

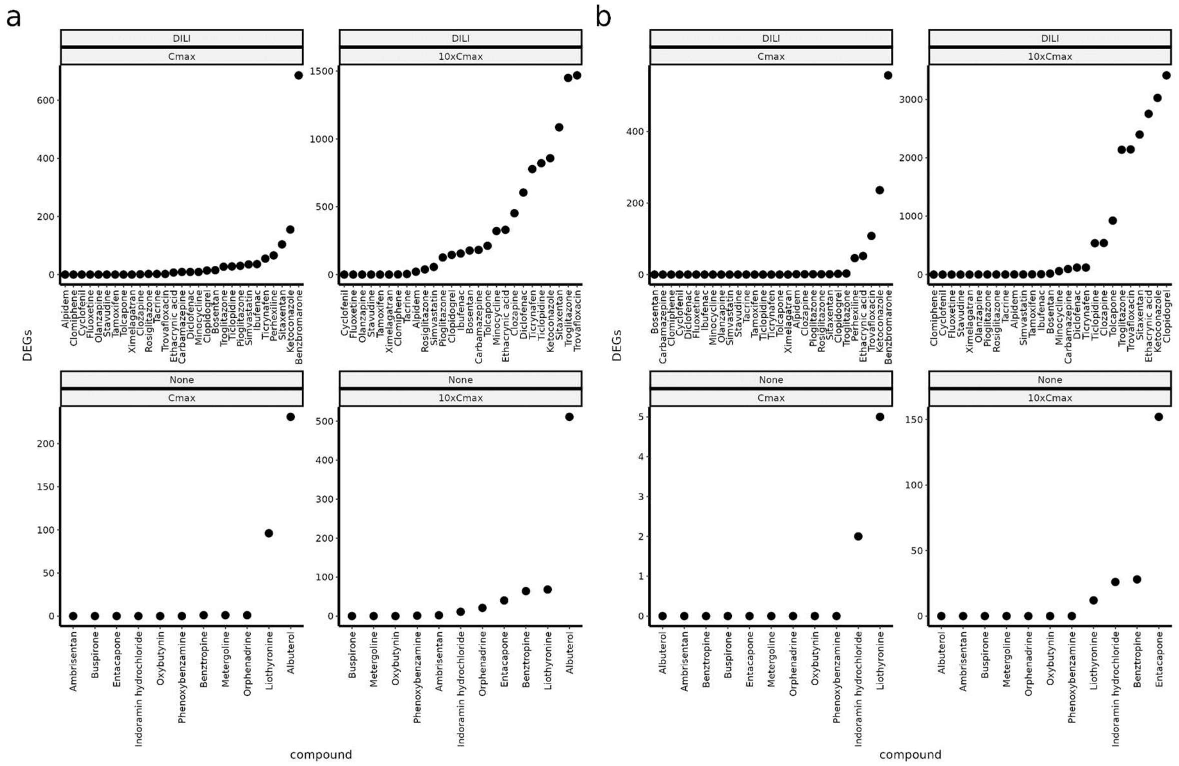

Figure 7. Overview of differentially expressed genes from DRUG-seq. Number of differentially expressed genes (adjusted pvalue <0.05) in (**a**) HepG2 cells and (**b**) PHH spheroids.

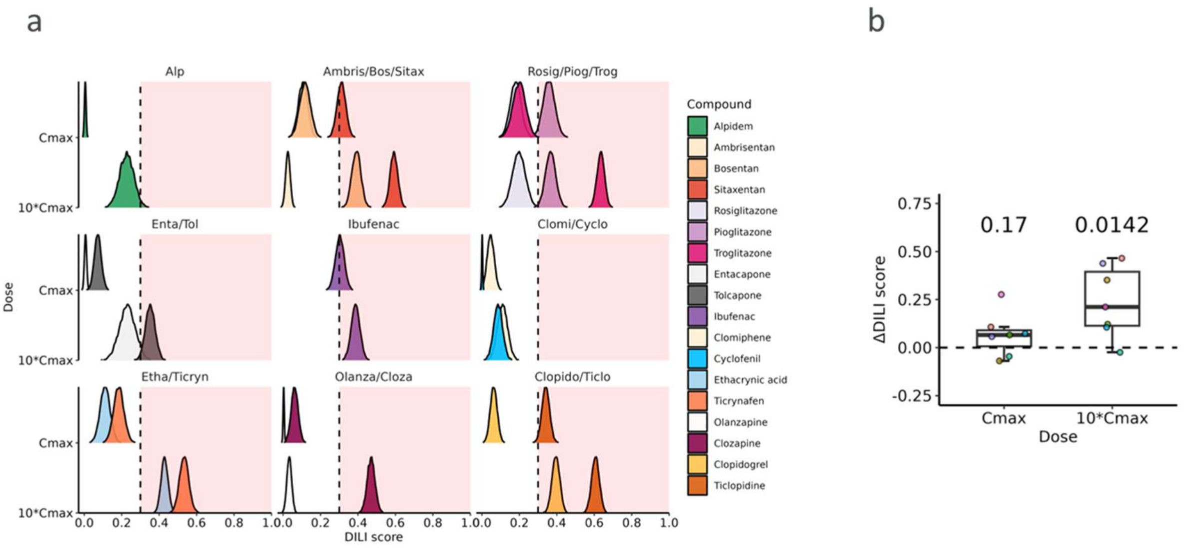

Figure 8. AEGIS DILI scores generated from DRUG-seq data from compound treatment in primary human hepatocyte spheroids. (**a**) Distribution of AEGIS DILI scores after running AEGIS 50 times and inserting noise into the expression data based on gene-level standard deviation across all samples. Darker colour corresponds to increasing Dose-Based DILI score from Chen et al. (2016) within a pair. The red area marks a high-risk zone with 89% confidence that a treatment is at high risk for DILI, see **Fig. 2b**. (**b**) Difference in DILI score (ΔDILI score) within 7 pairs of structurally similar compounds, where each pair contains one compound associated with more risk for DILI and the other compound with less risk for DILI.

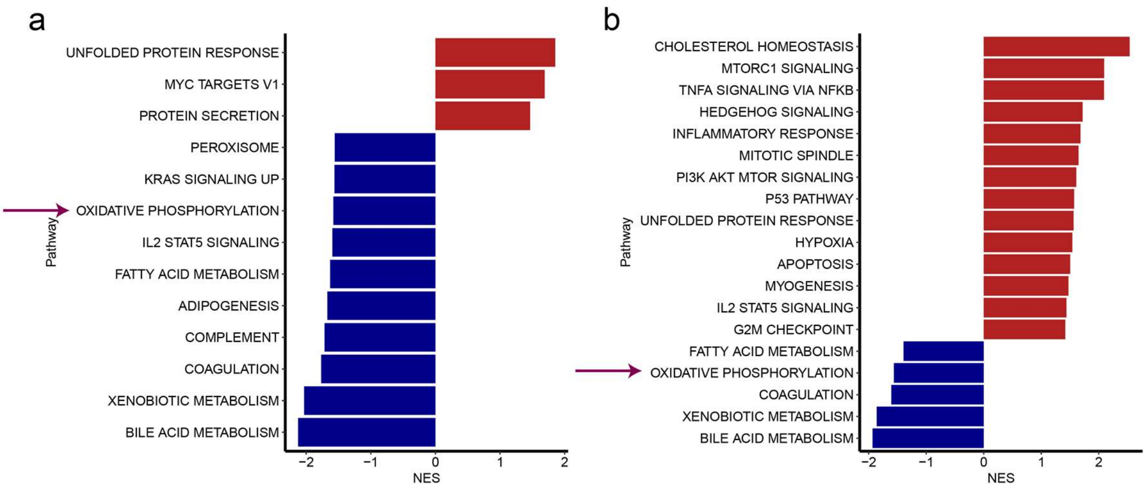

Figure 9. Functional analysis of AZD1979. Fast gene set enrichment analysis (fgsea) was used to assess the transcriptomics response of AZD1979 dosed at (**a**) C_{max} and (**b**) 10*C_{max} in primary human hepatocytes. The hallmark gene set collection was used, and only gene sets with significant normalized enrichment scores (NES) are shown (adjusted p-value <0.05)

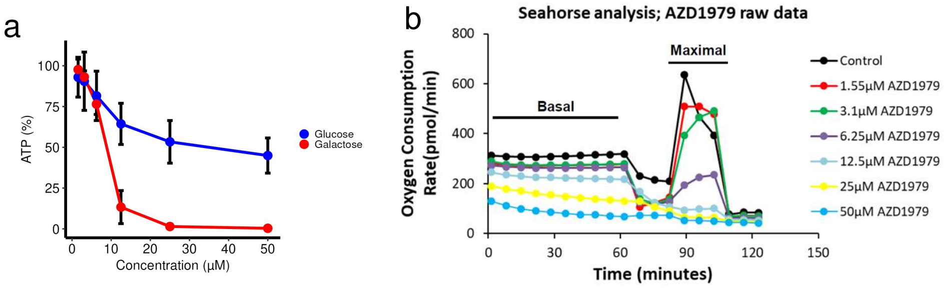

Figure 10. Mitochondrial toxicity assessment of AZD1979. (**a**) Changes in change in ATP production rate in glucose and galactose media relative baseline obtained from the Glu/Gal assay in HepG2 cells dosed with for 24 hours with AZD1979 (n = 3; error bars show the standard deviation). (**b**) Real-time oxygen consumption rates from the seahorse assay with different doses of AZD1979 after sequential addition of 1 µM oligomycin A, 1 µM FCCP and 5 µM antimycin A.

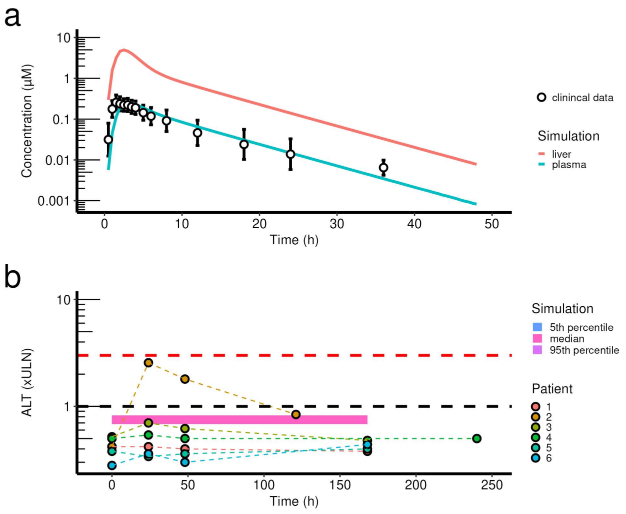

Figure 11. Liver exposure and ALT levels from 100 mg AZD1979. (**a**) PBPK simulation of plasma and liver concentrations of AZD1979 overlaid with clinical plasma levels of subjects that received 100mg dose of the drug. Clinical plasma levels are visualized as the geometric mean (n = 6; error bars show the geometric mean standard deviation). (**b**) Dynamic DILIsym simulation of ALT (normalized to ULN) showing the 5th percentile, median and 95th percentile of 285 individuals from a virtual population incorporating physiological variability of liver function, including ALT turnover and mitochondrial function. Clinical ALT values for six patients receiving 100 mg AZD1979 are shown as circles. Note: adverse event in patient 2 with ALT>1xULN was deemed causal and mild in severity. Black and red dashed line show 1*ULN and 3*ULN, respectively.

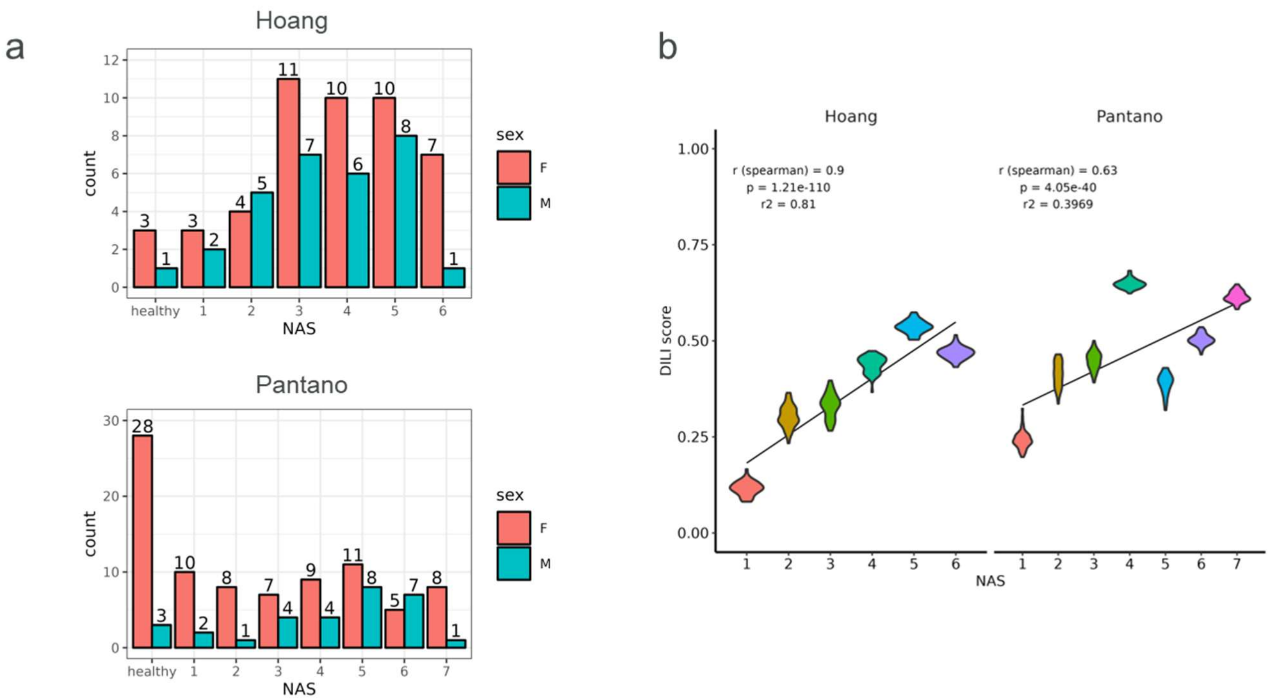

Figure 12. AEGIS DILI score on liver biopsy data from patients with increasing severity of MASLD. (**a**) Overview of samples and (**b**) the distribution of scores from 50 iterations of AEGIS with noise injection are visualized for each NAFLD activity score (NAS). Data from Hoang et al. (2019) and Pantano et al. (2021). Sex was used as a factor in the linear model in limma.

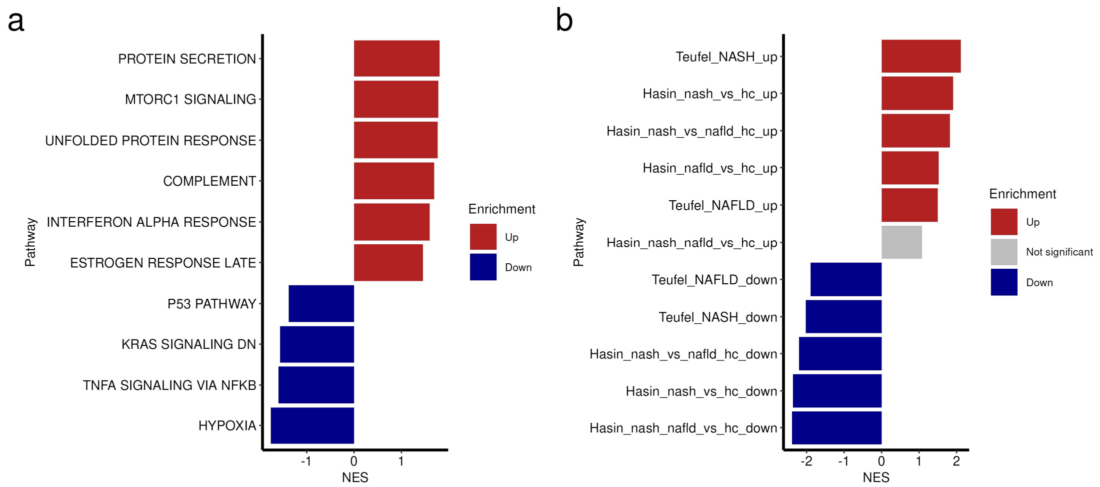

Figure 13. Evaluation of MASLD disease phenotype from transcriptomics data. Results from fast gene set enrichment analysis (fgsea) after comparing the gene expression between vehicle treatment of the MASLD and the healthy group. (**a**) Significant normalized enrichment scores (NES) from the hallmark gene set and (**b**) enrichment results from Hasin et al. (2022) and Teufel et al. (2016) signatures. A mixed effects model (implemented with duplicateCorrelation in limma) was used with sex and disease group as fixed effect, and donor as random effect, to generate the moderated t-statistics as input for fgsea.

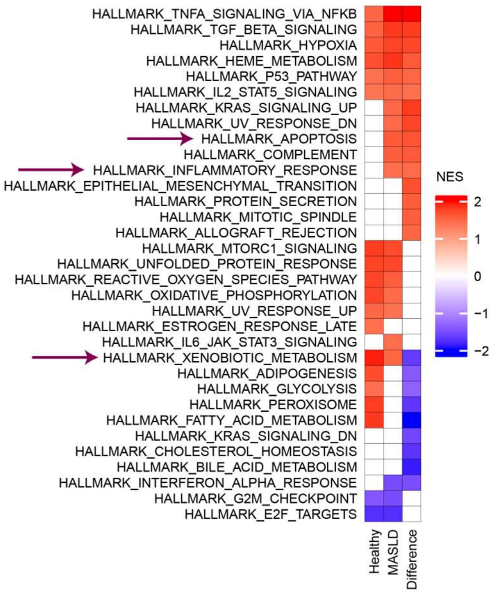

Figure 14. Evaluation of transcriptomics response in groups of healthy and MASLD donors. Results from fast gene set enrichment analysis (fgsea) after treatment with troglitazone at a concentration of 10*C_{max}, using the Hallmark gene set collection. The third column is the contrasted difference in response from troglitazone between both groups, after correcting for DMSO response. Gene sets with non-significant results (adjusted p-value >0.05) are set to 0. Gene sets marked with arrows are shown in **Fig. 5**.

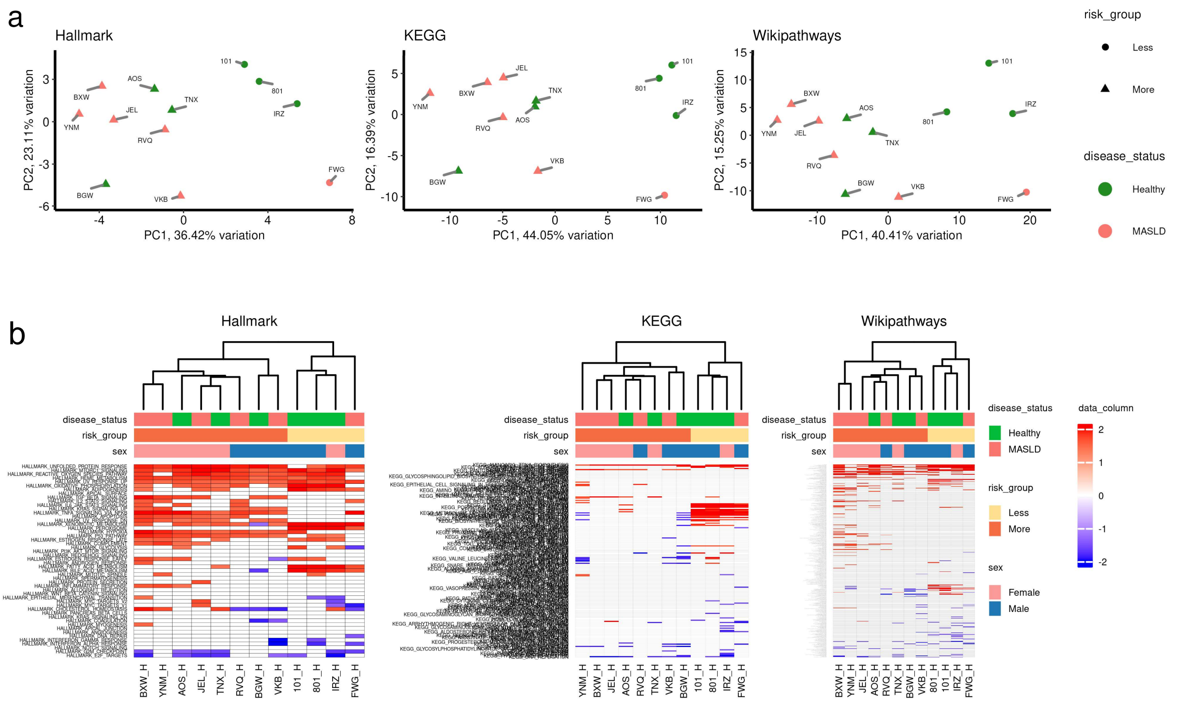

Figure 15. Identification of groups from donor specific effects. Groups were identified from the transcriptomic response from troglitazone treatment of 2D PHH isolated from healthy and MASLD patients. (**a**) PCA based on normalized enrichment score (NES) from fast gene set enrichment analysis (fgsea) with hallmark, KEGG and Wikipaths gene sets. (**b**) Significantly enriched NES (non-significant NES set to 0), with clustering based on all NES values.

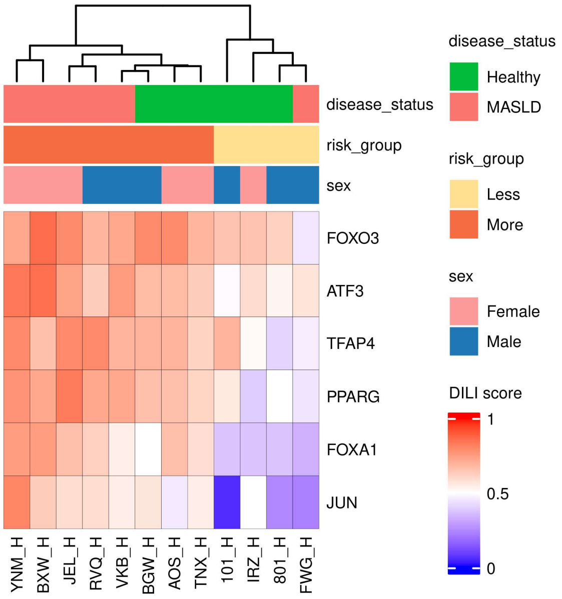

Figure 16. Donor-specific scores per AEGIS regulon. Donor-specific DILI scores per AEGIS regulon of 2D PHH isolated from healthy and MASLD patients treated with troglitazone Only AEGIS regulons with more than 15 genes present in the dataset were evaluated.

Supplementary Methods

Ethical Imperatives and Practices

AstraZeneca operate to the highest HBS ethical practices and ensure cell suppliers also adhere. BioIVT and Lifenet (hepatocyte suppliers) are committed to sourcing human biological specimens ethically and responsibly, following the regulatory practices, but additionally, the three guiding principles outlined in the Belmont Report, a foundational document written by the National Commission for the Protection of Human Subjects of Biomedical and Behavioral Research: respect for persons, beneficence, and justice. These principles are reflected in our guidelines as follows:

- **Informed Consent**: Obtaining informed consent from donors is vitally important. This includes clearly explaining the purpose of the research, procedures involved, potential risks and discomfort, and use of the HBS. Hepatocyte suppliers also disclose any alternative procedure or course of treatment that may be available and provide a point of contact for any follow-up questions. Donors must voluntarily agree to participate without any coercion and can decide to withdraw from the research at any point without repercussion. Additionally, donors are informed of potential commercial use for the research and that they will not receive any financial benefit from research results. Research using their HBS and data may also be included in scientific publications or presentations, but they will not be identified.
- **Transparency:** Researchers are transparent about how HBS will be used, stored, and shared. Hepatocyte suppliers include a statement of limited withdrawal in our informed consent documents, which explains that it may not be possible to destroy a participant's biospecimens and data upon request if those materials have already been released to other researchers.
- **Privacy and Confidentiality:** Protecting donor privacy and confidentiality is essential, so hepatocyte suppliers remove or code personal identifiers to prevent unauthorized access to that extremely sensitive information.
- **Regulatory Compliance:** Adhering to local, national, and international regulations is vital. This includes complying with data privacy laws such as HIPAA in the U.S. and GDPR in the UK and EU.
- **Ethical Review:** Research involving HBS undergo ethical review by either an AstraZeneca IRB or Independent Ethics Committee (IEC) to ensure that the rights and welfare of the donors are protected.

Filtering strategy for selecting the transcription factors use in AEGIS

- Collect transcription factors (TF) and gene targets from Dorothea
  - Non-academic version, evidence level A-C
- Collect GO-terms from AmiGO (https://amigo.geneontology.org/amigo)
- Keep
  - TFs with *positive regulation of apoptotic process* or its child terms
- Remove
  - TFs with *negative regulation of apoptotic process* or its child terms
  - TFs with evidence code *electronic annotation evidence* (IEA)
- Keep TFs with TPM >1 in liver according to GTEx Analysis V8 (https://gtexportal.org/home/downloads/adult-gtex/bulk_tissue_expression)
  - GTEx_Analysis_2017-06-05_v8_RNASeQCv1.1.9_gene_tpm.gct.gz

Table 1. Final list of transcription factors used in AEGIS.

TF	Apoptosis related GO-term	Evidence code	GO term reference	Liver TPM in GTEx	Number of target genes
ATF3	positive regulation of TRAIL-activated apoptotic signaling pathway	IMP	[1]	23.3	49
E2F3	positive regulation of vascular associated smooth muscle cell apoptotic process	IMP	[2]	2.25	12
FOXA1	positive regulation of apoptotic process	IDA	[3]	14.3	31
FOXO3	positive regulation of apoptotic process	IDA	[4]	7.8	34
JUN	positive regulation of apoptotic process	IMP	[5]	80.4	64
PPARG	positive regulation of vascular associated smooth muscle cell apoptotic process.	IMP	[6]	3.6	21
REST	positive regulation of apoptotic process	IMP	[7]	4.2	12
TFAP4	positive regulation of apoptotic process	IDA	[8]	3.9	40

Evidence codes: Inferred from Mutant Phenotype (IMP), Inferred from Direct Assay (IDA).

References

1. Edagawa M*, et al.* Role of activating transcription factor 3 (ATF3) in endoplasmic reticulum (ER) stress-induced sensitization of p53-deficient human colon cancer cells to tumor necrosis factor (TNF)-related apoptosis-inducing ligand (TRAIL)-mediated apoptosis through up-regulation of death receptor 5 (DR5) by zerumbone and celecoxib. *J Biol Chem* **289**, 21544-21561 (2014).
2. Gou D*, et al.* miR-210 has an antiapoptotic effect in pulmonary artery smooth muscle cells during hypoxia. *Am J Physiol Lung Cell Mol Physiol* **303**, L682-691 (2012).
3. Song L*, et al.* Role of Foxa1 in regulation of bcl2 expression during oxidative-stress-induced apoptosis in A549 type II pneumocytes. *Cell Stress Chaperones* **14**, 417-425 (2009).
4. Wang K, Li PF. Foxo3a regulates apoptosis by negatively targeting miR-21. *J Biol Chem* **285**, 16958-16966 (2010).
5. Imafuku I*, et al.* Presenilin 1 suppresses the function of c-Jun homodimers via interaction with QM/Jif-1. *J Cell Biol* **147**, 121-134 (1999).
6. Green DE*, et al.* Peroxisome proliferator-activated receptor-gamma enhances human pulmonary artery smooth muscle cell apoptosis through microRNA-21 and programmed cell death 4. *Am J Physiol Lung Cell Mol Physiol* **313**, L371-L383 (2017).
7. Lv H*, et al.* Expression and functions of the repressor element 1 (RE-1)-silencing transcription factor (REST) in breast cancer. *J Cell Biochem* **110**, 968-974 (2010).
8. Jung P, Menssen A, Mayr D, Hermeking H. AP4 encodes a c-MYC-inducible repressor of p21. *Proc Natl Acad Sci U S A* **105**, 15046-15051 (2008).
