# Supplementary material for: Apoptotic signatures allow early and rapid screening of drug-induced liver injury to accelerate drug discovery
Source: Commun Med (Lond). 2025 Dec 24;6:48. doi: 10.1038/s43856-025-01306-7 (PMC12827481; doi:10.1038/s43856-025-01306-7)
Supplement: Supplementary file 2 — Description of Additional Supplementary files [file 43856_2025_1306_MOESM2_ESM.docx]

**Description of Additional Supplementary Files**

Supplementary Data 1- Donor characteristics and compound information

Supplementary Data 2- The AEGIS gene signature

Supplementary Data 3- Source data for all main figures

Supplementary Code- AEGIS code example
